# E-PROOF: E-intervention for protein intake and resistance training to optimize function: A study protocol

**DOI:** 10.1371/journal.pone.0302727

**Published:** 2024-05-08

**Authors:** Jessica L. Krok-Schoen, Zachary L. Chaplow, Cara Chase, Colleen Spees, Ashley Rosko, Michelle J. Naughton, Jade Smith, Sam Soufi, Mike Beck, Brian C. Focht

**Affiliations:** 1 School of Health and Rehabilitation Sciences, College of Medicine, The Ohio State University, Columbus, OH, United States of America; 2 Department of Human Sciences, College of Education and Human Ecology, The Ohio State University, Columbus, OH, United States of America; 3 Department of Internal Medicine, College of Medicine, The Ohio State University, Columbus, OH, United States of America; PLOS: Public Library of Science, UNITED KINGDOM

## Abstract

**Background:**

Accounting for more than 60% of cancer survivors, older (≥65 years) cancer survivors have a 2- to 5-fold risk of physical function impairment, compared to cancer-free peers. One strategy to improve physical function is dietary and resistance training interventions, which improve muscle strength and mass by stimulating muscle protein synthesis. The E-PROOF (**E**-intervention for **P**rotein Intake and **R**esistance Training t**o O**ptimize **F**unction) study will examine the feasibility, acceptability, and preliminary efficacy of a 12-week randomized controlled trial of an online, tailored nutritional and resistance training education and counseling intervention to improve physical function and associated health outcomes (muscle strength, health-related quality of life (HRQoL), self-efficacy, and weight management).

**Methods:**

In this study, 70 older cancer survivors will be randomized to one of two groups: experimental (receiving remote behavioral counseling and evidence-based education and resources), and control (general survivorship education). We will examine the intervention effects on physical function, muscle strength, HRQoL, self-efficacy, weight, and waist circumference during a 12-week period between the experimental and control groups. Three months following the end of the intervention, we will conduct a follow-up assessment to measure physical function, muscle strength, and HRQoL.

**Significance and impact:**

This study is the first synchronous, online protein-focused diet and resistance training intervention among older cancer survivors. This novel study advances science by promoting independent health behaviors among older cancer survivors to improve health outcomes, and provide foundational knowledge to further address this growing problem on a wider scale through online platforms.

## Introduction

The older cancer survivor (OCS) population (≥65 years) is comprised of ≥12 million adults [1], and is increasing rapidly due to advances in cancer screening and treatment and the aging of the U.S. population [2]. Despite accounting for 67% of cancer survivors [3], OCS are vastly understudied in survivorship research [2,4,5]. Additive effects of cancer treatments, age-related musculoskeletal and soft-tissue degeneration, and increased comorbidity result in a 2- to 5-fold increased risk of functional limitations among OCS, compared to cancer-free controls [6–10]. Further, recent longitudinal studies found OCS have accelerated declines in muscle mass, evidenced by worsening grip strength, slower gait speed, and lower overall performance than older adults without cancer [6,11,12]. Physical function impairment is associated with a myriad of poor health outcomes including diminished health-related quality of life (HRQoL), multimorbidity, increased falls, and all-cause mortality [13–16]. Although a loss of muscle mass and decline in physical function may be expected with aging, there is variation in the decline, suggesting modifiable behaviors (diet, exercise) could influence physical function [17,18]. Improving physical function among this vulnerable group increases the ability to live independently, which reduces burden to themselves, loved ones, and the healthcare system [19,20]. Thus, addressing physical function among OCS is an immediate and increasing need [21–23].

Recent studies have found that physical needs including diet and exercise are the highest reported unmet needs, and physical challenges (i.e., loss of strength) were the most frequently reported problems among OCS [24,25]. Yet, most survivors receive minimal, if any, diet and exercise counseling as part of their cancer care [25–27]. This lack of tailored guidance on positive health behaviors [28] to mitigate these issues necessitates immediate intervention.

Substantial evidence from randomized trials supports the potential for *combined* exercise and diet interventions to improve physical function among older adults and cancer survivors [7,22,29–33]. Diet and exercise interventions can improve function through stimulating muscle protein synthesis, which increases muscle strength and mass. Dietary protein supplementation is required to maximize skeletal muscle mass gain during prolonged resistance-type exercise training, which more effectively counteracts sarcopenia and physical function decline [34–37]. Despite this mounting evidence, there is a paucity of research focusing on protein consumption and resistance training among OCS, a group at high risk for functional limitations [38]. Few studies have used resistance exercise as the main mode of training [39–43], despite its established association with improved physical function among older adults [44–47]. Further, a recent study found resistance exercise improved more outcomes than aerobic exercise, including muscle strength, aerobic capacity, and physical function among older breast cancer survivors [48].

Previous research demonstrates the acceptability and effectiveness of technology (e.g., Fitbits, apps) to improve health outcomes (exercise, balance, HRQoL) of OCS [48,49]. Prior work recommends one-on-one, synchronous online conversations as the most effective approach for older adults [50–52], providing real-time guidance and support. Considering the influx of telehealth due to COVID-19, increased technology use among older adults (84% own a cellphone, computer, or tablet and know how to use it [53]), and the positive impact of online behavior change interventions for cancer survivors [49,54–58], online-based interventions to improve physical function need to be tested within OCS, a vulnerable and growing population. Until our study, there were no synchronous, online diet and exercise interventions for OCS. The E-PROOF (E-intervention for Protein Intake and Resistance Training to Optimize Function) study (1R21AG078258-01A1) will implement and assess the efficacy of the first online dietary and resistance training intervention to improve OCS’ physical function and improve associated health outcomes (muscle strength, dietary quality, health-related quality of life, self-efficacy, weight management).

## Materials and methods

### Aim, design, and setting

**Fig 1:**
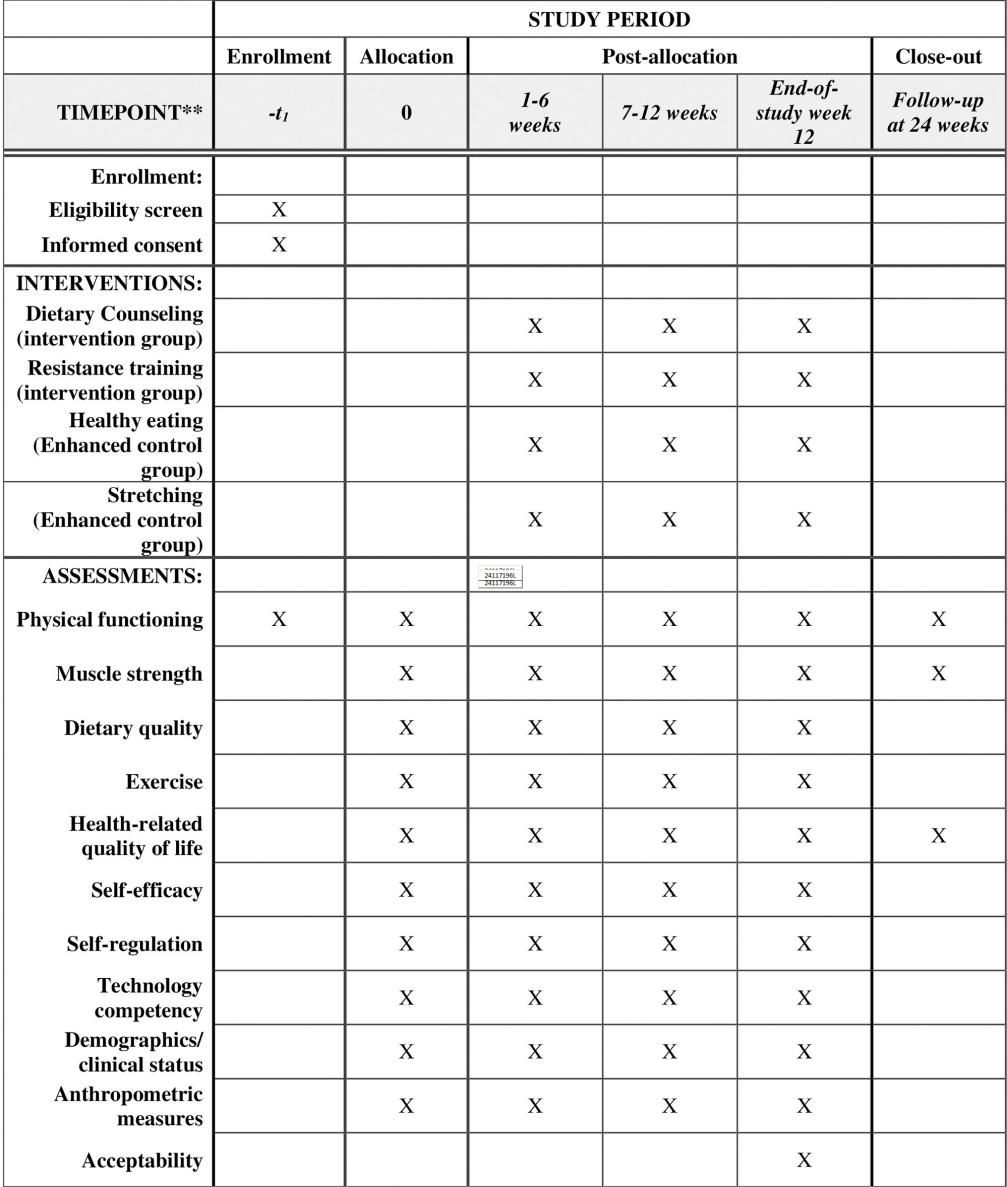
SPIRIT schedule of enrollment, interventions, and assessments for EPROOF study.

**Fig 2:**
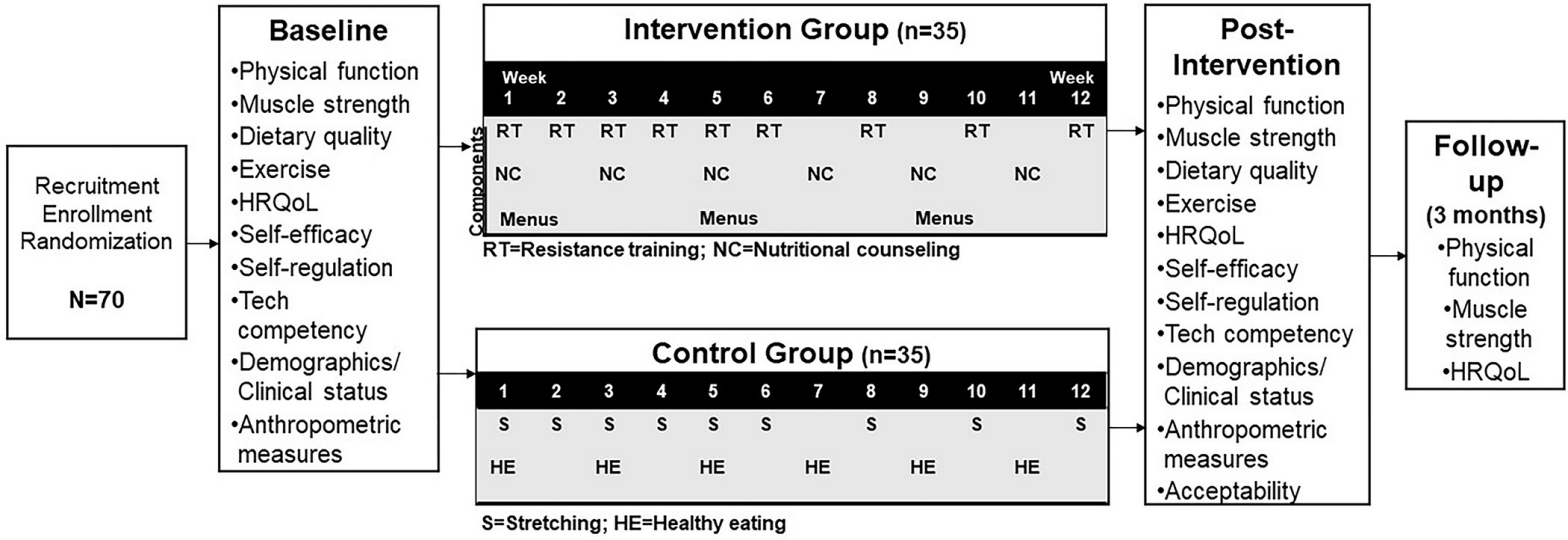
EPROOF study schema.

The EPROOF study is a 12-week pilot, randomized controlled trial (See SPIRIT [59] Fig 1; study schema Fig 2). This study’s overall objective is to improve physical function through online, tailored nutritional and resistance training counseling to increase protein intake, improve diet quality, increase resistance training, and improve related health outcomes (muscle strength, dietary quality, HRQoL, self-efficacy, weight management). In this pilot randomized controlled trial, participants (n = 70) will be randomized to one of two groups: experimental (online counseling, menus, educational materials) and enhanced control (passive educational materials). We will examine the intervention effects on physical function and associated health outcomes during the 12-week period between the experimental and control group. This ongoing study is taking place at The Ohio State University, specifically within the Physical Activity and Education Sciences lab, on the main campus.

### Sample size, eligibility criteria, and recruitment

The target sample size is 70 older cancer survivors (n = 35 for intervention arm, n = 35 for control arm). For this study, the minimum sample size is calculated using G*Power. Specifying alpha level to be 0.05 (one-sided), expected power to be 0.80, and an expected effect size of 0.50 for the primary outcome (physical function measured by Short Physical Performance Battery (SPPB) [60]), the sample size required is 27 per group. By recruiting 70 participants, we allow for a 22% attrition rate.

Eligibility criteria are: ≥65 years of age, a primary diagnosis of stage I-III breast, colorectal, or prostate cancer, completion of primary curative treatment, reported at least 1 physical function limitation on the 10-question RAND-36 Physical Function Subscale (PFSS) [61] (“limited a little”, “limited a lot”), no evidence of progressive disease or second cancers, community-dwelling, and able to provide written informed consent. Individuals are excluded if they currently receive cancer treatment (e.g., chemotherapy, radiation), have liver and/or renal disease limiting their protein intake, are under the care of a registered dietitian (RD)/nutritionist, participating in other diet/exercise interventions, consume protein supplements, and have contraindications to unsupervised exercise (e.g., walker/wheelchair use).

Recruitment began on August 28, 2023, and occurs through several methods. The first method is through previously consented older cancer survivors in the Ohio State University Comprehensive Cancer Center’s Total Cancer Care (TCC) registry. The TCC registry, a personalized cancer care initiative designed to collect clinical data and tumor specimens throughout a patient’s lifetime, will be utilized to capture a larger cancer population. Based on the eligibility criteria, TCC will contact potential participants, identified across all OSUCCC clinics, through phone calls, to introduce the proposed study and see if they agree to be contacted by the EPROOF study staff. Second, is through advertisements on Facebook and ResearchMatch^©^, a free and secure online tool created by academic institutions across the country to share study and PI contact information to potentially eligible participants. Third, is through flyers distributed to potentially eligible participants at the OSUCCC’s Cancer and Aging Resiliency clinics, site-specific medical oncology clinics (breast, colorectal, and prostate) and Survivorship clinics. Lastly, through community engagement including presentations at Columbus-area senior centers, health-oriented community groups, and cancer-specific non-profit organizations. All these methods ask potential participants interested in the study to contact the clinical research coordinator to ask questions and complete the screening questionnaire to confirm eligibility.

### Randomization

Subjects are block randomized by the number of physical function limitations measured by the PFSS [62]. Randomization to study group (intervention, enhanced control) occurs prior to the baseline assessment, via an online random team generator tool used by the clinical research coordinator. Potential participants are informed of the chance of randomization during the eligibility screening. Equitable distribution of participants between the intervention and attentional control group occurs as needed throughout study recruitment. Participants are blinded to their treatment group to reduce potential bias and foster engagement to the same extent, regardless of group.

### Intervention

The study consists of three in-person clinic visits for participants in both study arms. At the baseline visit, study staff will attain written informed consent and measure the physical function (SPPB), muscle strength (handgrip, SPPB), height, weight, and waist circumference of all participants. Online questionnaires, via Research Electronic Data Capture (REDCap), will assess dietary quality, self-reported exercise, HRQoL, self-efficacy, and demographic and clinical information. After data collection, the research assistant will show intervention participants how to access the REDCap-based logs and enter their weekly dietary intake and exercise adherence. At the end of the baseline visit, intervention participants will receive a binder with printed educational materials on increasing dietary protein, healthy eating, and resistance training to preserve or rebuild lean body mass as a reference tool. Resistance bands (three levels of Thera-bands®), and resistance band exercise guidelines (exercises by muscle group, proper form, how to rate difficulty) will be distributed to intervention participants. Enhanced control group participants receive printed materials (National Cancer Institute’s “Facing Forward: Life After Cancer Treatment”) pertaining to general cancer survivorship.

Social Cognitive Theory [63] and the Health Belief Model [64] provide a foundation for behavior changes (increased protein intake, improved diet quality, and increased resistance exercise) proposed in this intervention. Motivational interviewing strategies support the participants’ progress, provide reinforcement, explore strategies to overcome barriers, field questions, and establish tailored goals.

#### a. Intervention group

Within 1 week of the baseline visit, an RD will review the dietary quality results and create a tailored dietary plan focusing on protein intake and healthy eating for each intervention participant. At weeks 1, 3, 5, 7, 9, and 11, the RD will provide one-on-one, online nutritional counseling to the intervention participants. Counseling will consist of education to stimulate sufficient protein- and energy intake using regular food, based on the current American Cancer Society nutrition guidelines for cancer survivors [65] and DGAs for protein and energy for adults aged ≥60 (5–6.5 oz. eq/day based on 1,600–2,600 calories/day) [66].

For the first 6 weeks, the exercise scientist (ES) will host weekly, one-on-one, 30-minute, online resistance training sessions for the intervention group, using a progression model [67]. After week 6, the resistance training sessions will taper, and be held biweekly at weeks 8, 10, and 12. In each training session, the ES will provide education, then lead participants through a 5-minute warm-up, followed by a series of whole-body exercises (each major muscle group) performed at each session, and a 5-minute cool-down. The training is based on the ACSM exercise guidelines for cancer survivors [68] and ACSM recommendations for progressive resistance training for older adults [69] consisting of 1–3 sets of 8–10 exercises with 8–12 repetitions with a 1–2 min rest between sets. Participants will be encouraged to complete these exercises two more times/week, totaling three sessions/week. Intervention group participants will be asked to complete weekly online diaries of their weekly diet and exercise.

#### b. Enhanced control group

The enhanced control group will receive general healthy eating sessions (RD) and whole body stretching (ES) to match the attention provided to the intervention group. The RD will provide 30-minute online information sessions at weeks 1, 3, 5, 7, and 9. The ES will hold online stretching sessions for 30 minutes in weeks 1–6, then in weeks 8, 10, and 12. These control group sessions will not educate on or promote protein intake, resistance training, and physical function. Enhanced control group participants are also asked to complete weekly online diaries of their weekly diet and exercise.

### Outcomes and measures

The measures used in the EPROOF study are validated within older adults and/or older cancer survivors and used within prior lifestyle interventions with cancer survivors [7,46,49,70,71].

Primary outcome: Physical Function. The physical performance assessment, SPPB [60], will measure physical function at baseline, end-of-study, and follow-up. The SPPB includes three lower extremity physical performance measures (standing balance, five consecutive chair rises, 4-meter gait walk at usual pace) to assess lower extremity strength. Summary scores range from 0 to 12, with higher scores denoting higher physical function.

Secondary outcomes:

#### Acceptability and feasibility

At end-of-study, intervention group participants will complete a 5-item, Likert-scale questionnaire, with written comments, regarding their preferences for receiving education and counseling, successes and challenges, program satisfaction, and suggestions for improvement. Intervention feasibility will be measured prospectively tracking rates of accrual, retention, and adherence at baseline, end-of-study, and 3-month follow-up. Recruitment rate is based on Consolidated Standards of Reporting Trials criteria [72] that includes eligible consented individuals and eligible non-consented individuals with non-recruitment reasons documented. Process information (number/duration of video visits) will be recorded.

#### Muscle strength

Handgrip strength will be measured at baseline, end-of-study, and follow-up in both hands using a hydraulic grip strength dynamometer (Jamar Model 7498). Three trials with brief pauses will be conducted for each hand and the mean score of the six trials will be used as the mean handgrip score.

#### Dietary quality

Protein intake and dietary quality will be assessed at baseline and end-of-study by the National Cancer Institute’s Diet History Questionnaire III (DHQIII) [73]. The DHQIII assesses Healthy Eating Index-2015 (HEI-2015) total and individual component scores (e.g., protein) and average daily nutrient intakes, according to the DGA [74].

#### Resistance exercise

Self-reported resistance exercise will be assessed using the revised version of The Godin-Shephard Leisure-Time Exercise Questionnaire modified to specifically capture self-reported resistance exercise participation. This measure has been used extensively to capture self-reported resistance exercise in past observational and interventional trials [71,75–77].

#### HRQoL

RAND-36 Health Status Measure [61] is comprised of 8 subscales assessing multiple aspects of HRQoL. Physical and mental component summaries are created. All scores range from 0–100, with 100 as the highest. Changes of ≥5 points on the component scores are considered clinically relevant [78,79]. The RAND-36 will be completed at baseline, end-of-study, and follow-up.

#### Self-efficacy

Baseline and end-of-study self-efficacy for diet and resistance training will be measured by: “How sure are you that you could do exercises to make your body stronger for 15 minutes, 3 days a week?” and “How sure are you that you could improve your diet?” A 10-point Likert response scale, ranging from a score of 1 (completely uncertain) to 10 (completely certain), will be used.

#### Self-regulation

Baseline and end-of-study self-regulation for diet and exercise will be measured by the 12-item Dietary Self-Regulation Scale [70] and the 12-item Exercise Self-Regulation Scale [80].

#### Technology competency

The 8-item eHealth Literacy Scale (eHEALS) [81] will measure knowledge, comfort, and perceived skills of engaging in eHealth at baseline and end-of-study. A 5-point Likert scale will be used.

#### Demographic and health information

Participants’ age, race, ethnicity, sex, education, income, marital status, insurance status, cancer diagnosis, stage at diagnosis, time since diagnosis, treatments received (e.g., chemotherapy, radiation) and comorbidities will be collected at baseline and updated at end-of-study. Body weight, height, and waist circumference will be collected at baseline and end-of-study.

### Retention

The following is a brief point summary of guidelines to promote retention of participants and adherence to the protocol: Fostering personal participant-staff relationships; Maintaining continuity of care; Maintaining an inviting, convenient, and accessible environment for in-person data collection; Consistent and clear participant-staff communications; Minimizing wasted time during in-person data collection; Appointment reminders by mail and telephone; Providing contact information (phone number, email address) of the clinical research coordinator and study PI. Lastly, based on feedback from the patient advocacy group within the Cancer and Aging research group, intervention participants will also have the option of attending Zoom^®^-based social hours to share their thoughts and experiences with each other. This socialization opportunity may also promote retention, motivation, and adherence among intervention participants.

### Data management

The proposed intervention utilizes an individualized approach and does not operate in group settings, therefore ensuring participants’ privacy. All data points will be collated and stored on a REDCap database, a secure web application [82]. Data will be used only in aggregate and no identifying characteristics of individuals will be published or presented. Confidentiality of data will be maintained by using research identification numbers that uniquely identify each individual. Data other than demographic information do not use names as an identifier. Safeguards will be established to ensure the security and privacy of participants’ study records. All computer systems will be password-protected against intrusion. All network-based communications of confidential information will be encrypted.

All paper files and computer files with the de-identified data will be stored under lock and key at all times. The files matching participants’ names and demographic information with research numbers will be kept in a separate room and will be stored in a locked file that uses a different key from that of all other files. Only study personnel will have access to these files, and they will be asked to sign a document that they agree to maintain the confidentiality of the information.

### Safety considerations

We anticipate that this study will entail minimal physical and psychological risks for study participants. There is a small risk to participants of sustaining an injury while participating in the intervention. Physical injury is not anticipated, and the trained ES will provide guidance on how to participate in resistance training, safely and effectively. Participant risk may also include, but is not anticipated, psychological distress associated with completion of the online questionnaires. If this occurs, study staff will speak to the participant about the difficulties that the questionnaires have caused and will offer a referral to a mental health professional, if necessary. Lastly, we are proposing a synchronous online intervention design to reduce psychological distress and assist with the maintenance of health behaviors among participants.

In order protect against risk, all study staff are trained on the unique needs and challenges of older cancer survivors prior to the intervention. Participants are also screened for physical limitations prior to enrollment. If a potential participant has three or more reported physical limitations, we will obtain a release form from each participant’s primary care physician for involvement in the study. We also will refer to the project’s safety officer when we have questions about potential safety risks as well as any injury or symptoms due to study participation. Lastly, provision of the educational materials, video-based synchronous dietary and exercise sessions, and participant logs, all provide methods of addressing potential participant difficulties and thus, reducing risk.

### Planned analyses

Analyses will be conducted by a PhD-level statistician. The feasibility of implementing a 12-week online diet and resistance training intervention with OCS will be determined by the study accrual rate, retention rate, and adherence rate. The study accrual rate will be calculated by dividing the number of potential participants that passed screening by the total number of participants started in the study after appropriate informed consent procedures. The recruitment goal is set at 70 participants recruited over a 9-month period. Retention rate will be calculated by dividing the total number of participants initiated by total number of participants in the study at baseline and 12 weeks (end-of-study). The retention goal for this trial is 80%. If 80% (56/70) of participants are retained at end-of-study, the retention goal for this trial will be achieved. The primary outcome of the SPPB will be assessed at three time points: baseline, end-of-study, and 3-month follow-up. Efficacy will be based on 12 weeks.

The SPPB score is a continuous variable, collected as the total score ranging from 0–12. Group differences in SPPB scores over time will be tested using repeated measures ANOVA (SAS PROC MIXED). Using the planned contrasts command, we will investigate the changes in SPPB scores within each group, and between the intervention and control groups at end-of-study. Descriptive statistics and frequency distributions will be used to characterize the sample. To examine the effect of the intervention on the changes in physical function, a linear mixed effect model will be adopted using the SAS PROC MIXED procedure. The intervention group (intervention vs. control) and time points (baseline, end-of-study, 3-month follow-up) serve as the categorical predictors, together with the intervention*time interaction term. A significant intervention*time interaction effect would indicate a differential change pattern over the time between the groups. Using post-hoc contrasts, we will compare group differences in collected variables at each time point. Using planned contrasts command, we will investigate for each group the change from baseline to end-of-study, from end-of-study to 3-month follow-up, and from baseline to 3-month follow-up to capture the change patterns over the phases and Bonferroni correction will be applied. A Shapiro-Wilk test, skewness, kurtosis, and Q-Q plots will be used to assess normality of survivor baseline and end-of-study outcomes. Based on normality tests, change in participant outcome measures from baseline to end-of-study will be assessed using paired t-tests or Wilcoxon’s signed rank tests. Analysis will be carried out using intent-to-treat (all participants) and modified intent-to-treat (adherent participants only). All quantitative analyses will be performed using SAS version 9.4.

### Ethical considerations and declarations

All study materials and protocols have been reviewed and approved by the OSUCCC’s Clinical Scientific Review Committee and OSU’s Institutional Review Board (2022C0220). This study is registered on at the National Institutes of Health’s clinical trials website (NCT06016725).

### Status and timeline

The study is currently enrolling participants. Participant recruitment began at the end of August 2023 and will continue into summer 2024. End-of-study visits and subsequent analyses will be completed by early 2025 and May 2025, respectively.

## Discussion

### Limitations

There are some limitations to this study. First, potential participants must have and be able to utilize web-based computers or smartphones. This may inadvertently exclude individuals from enrolling in the study if they do not have these resources. Another limitation is that despite this intervention being mostly online, there are three in-person physical assessments, which can decrease the potential participant population. However, we have found that participants are willing to drive up to approximately 100 miles to participate. The last limitation is the limited generalizability of this study because OCS are recruited from one academic medical center and the greater Columbus, Ohio area. Efforts to recruit a diverse participant group include multiple recruitment strategies and less restrictive eligibility criteria.

### Dissemination and future directions

We will present the initial and final research findings at scientific conferences related to cancer survivorship, health behavior, geriatric oncology, and geriatrics. We will upload our abstracts and presentations to be made public when prompted by the conference organizers. The study team will also submit manuscripts to peer-reviewed journals in the fields of cancer survivorship, health behavior, geriatric oncology, and geriatrics. The research team will submit manuscripts to peer-reviewed open access journals and/or opt-in for open access when provided the option. We also will deposit our data through the National Archive of Computerized Data on Aging (NACDA), which is a NIH-funded repository. These data will be shared with investigators working in an institution with a Federal Wide Assurance (FWA) and can be used for secondary study purposes, such as finding disparities in survivorship outcomes.

The proposed study will significantly contribute to our understanding of diet and resistance training interventions among older cancer survivors. This work will provide greater insight into how protein and diet quality can mitigate physical function impairment, knowledge that is currently lacking among older cancer survivors, and that promises to yield novel insights into aging, lifestyle behaviors, and cancer survivorship. The results of this study will be used as the foundation for a future R01 randomized controlled trial to examine the impact of a protein-focused meal delivery program and resistance exercise regimen on physical function and sarcopenia among older cancer survivors, as determined by body composition imaging and physical performance test batteries. We will explore additional questions through mixed methods observational research to understand motivation and the role of social support in diet and exercise adoption and adherence among OCS.

## Supporting information

S1 File (DOCX)

## References

[1] Siegel RL, Giaquinto AN, Jemal A. Cancer statistics, 2024. CA: A Cancer Journal for Clinicians. 2024;74(1):12–49. doi: 10.3322/caac.21820 38230766

[2] Bluethmann SM, Mariotto AB, Rowland JH. Anticipating the "Silver Tsunami": Prevalence, trajectories and comorbidity burden among older cancer survivors in the United States. Cancer Epidemiology, Biomarkers & Prevention. 2016;25(7):1029–36. doi: 10.1158/1055-9965.EPI-16-0133 27371756 PMC4933329

[3] Miller KD, Nogueira L, Devasia T, Mariotto AB, Yabroff KR, Jemal A, et al. Cancer treatment and survivorship statistics, 2022. CA: A Cancer Journal for Clinicians. 2022;n/a(n/a):in press. 10.3322/caac.21731 35736631

[4] Aziz NM. Cancer survivorship research: state of knowledge, challenges and opportunities. Acta oncologica. 2007;46(4):417–32. doi: 10.1080/02841860701367878 17497308

[5] DuMontier C, Driver JA. Advancing Survivorship in Older Adults With Cancer. Oxford University Press; 2021. p. 1444–6. 10.1093/gerona/glab126 PMC8277077 34156074

[6] Williams GR, Chen Y, Kenzik KM, McDonald A, Shachar SS, Klepin HD, et al. Assessment of sarcopenia measures, survival, and disability in older adults before and after diagnosis with cancer. JAMA Network Open. 2020;3(5):e204783–e. doi: 10.1001/jamanetworkopen.2020.4783 32396194 PMC7218493

[7] Kenzik KM, Morey MC, Cohen HJ, Sloane R, Demark-Wahnefried W. Symptoms, weight loss, and physical function in a lifestyle intervention study of older cancer survivors. Journal of Geriatric Oncology. 2015;6(6):424–32. doi: 10.1016/j.jgo.2015.08.004 26362355 PMC4662250

[8] Cohen HJ. Keynote comment: cancer survivorship and ageing—a double whammy. The Lancet Oncology. 2006;7(11):882. doi: 10.1016/S1470-2045(06)70913-5 17081910

[9] Leach CR, Bellizzi KM, Hurria A, Reeve BB. Is it my cancer or am I just getting older?: Impact of cancer on age-related health conditions of older cancer survivors. Cancer. 2016;122(12):1946–53. doi: 10.1002/cncr.29914 27159822

[10] Chavan P, Kedia S, Yu X. Physical and functional limitations in US older cancer survivors. Journal of Palliative Care & Medicine. 2017;7(4).

[11] Siddique A, Simonsick EM, Gallicchio L. Functional decline among older cancer survivors in the Baltimore longitudinal study of aging. Journal of the American Geriatrics Society. 2021;69(11):3124–33. doi: 10.1111/jgs.17369 34346072 PMC8595548

[12] Luo J, Carter SJ, Feliciano EMC, Hendryx M. Trajectories of objectively measured physical function among older breast cancer survivors in comparison with cancer-free controls. Breast Cancer Res Treat. 2022. doi: 10.1007/s10549-022-06568-6 35347550 PMC9173672

[13] Koll TT, Semin JN, Brodsky R, Keehn D, Fisher AL, High R, et al. Health-related and sociodemographic factors associated with physical frailty among older cancer survivors. Journal of Geriatric Oncology. 2021;12(1):96–101. doi: 10.1016/j.jgo.2020.04.015 32451313 PMC7680287

[14] Studenski S, Perera S, Patel K, Rosano C, Faulkner K, Inzitari M, et al. Gait speed and survival in older adults. JAMA. 2011;305(1):50–8. doi: 10.1001/jama.2010.1923 21205966 PMC3080184

[15] Brown JC, Harhay MO, Harhay MN. Physical function as a prognostic biomarker among cancer survivors. British journal of cancer. 2015;112(1):194–8. doi: 10.1038/bjc.2014.568 25393366 PMC4453612

[16] Morishita S, Tsubaki A, Fu JB, Mitobe Y, Onishi H, Tsuji T. Cancer survivors exhibit a different relationship between muscle strength and health-related quality of life/fatigue compared to healthy subjects. European Journal of Cancer Care. 2018;27(4):e12856. doi: 10.1111/ecc.12856 29767832

[17] Duan-Porter W, Cohen HJ, Demark-Wahnefried W, Sloane R, Pendergast JF, Snyder DC, et al. Physical resilience of older cancer survivors: An emerging concept. J Geriatr Oncol. 2016;7(6):471–8. doi: 10.1016/j.jgo.2016.07.009 27478133 PMC5159214

[18] Bloom I, Shand C, Cooper C, Robinson S, Baird J. Diet quality and sarcopenia in older adults: a systematic review. Nutrients. 2018;10(3):308. doi: 10.3390/nu10030308 29510572 PMC5872726

[19] Williams GR, Dunham L, Chang Y, Deal AM, Pergolotti M, Lund JL, et al. Geriatric assessment predicts hospitalization frequency and long-term care use in older adult cancer survivors. Journal of Oncology Practice. 2019;15(5):e399–e409. doi: 10.1200/JOP.18.00368 30870086 PMC7846045

[20] Brown JC, Harhay MO, Harhay MN. Patient-reported versus objectively-measured physical function and mortality risk among cancer survivors. Journal of Geriatric Oncology. 2016;7(2):108–15. doi: 10.1016/j.jgo.2016.01.009 26907563 PMC4818688

[21] Shahrokni A, Wu AJ, Carter J, Lichtman SM. Long-term toxicity of cancer treatment in older patients. Clin Geriatr Med. 2016;32(1):63–80. doi: 10.1016/j.cger.2015.08.005 26614861 PMC4839483

[22] Mustian K, Lin P-J, Cole C, Loh KP, Magnuson A. Exercise and the older cancer survivor. In: Extermann M, editor. Geriatric Oncology. Cham: Springer International Publishing; 2020. p. 917–38.

[23] Deimling GT, Pappada H, Ye M, Nalepa E, Ciaralli S, Phelps E, et al. Factors affecting perceptions of disability and self-rated health among older adult, long-term cancer survivors. Journal of aging and health. 2019;31(4):667–84. doi: 10.1177/0898264317745745 29254449

[24] Burg MA, Adorno G, Lopez ED, Loerzel V, Stein K, Wallace C, et al. Current unmet needs of cancer survivors: Analysis of open‐ended responses to the American Cancer Society Study of Cancer Survivors II. Cancer. 2015;121(4):623–30. doi: 10.1002/cncr.28951 25581252

[25] Fitch MI, Nicoll I, Lockwood G, Strohschein FJ, Newton L. Main challenges in survivorship transitions: Perspectives of older adults with cancer. Journal of Geriatric Oncology. 2020. doi: 10.1016/j.jgo.2020.09.024 33008768

[26] Krok-Schoen JL, Naughton MJ, Noonan AM, Pisegna J, DeSalvo J, Lustberg MB. Perspectives of survivorship care plans among older breast cancer survivors: A pilot study. Cancer control: journal of the Moffitt Cancer Center. 2020;27(1):1073274820917208. doi: 10.1177/1073274820917208 32233798 PMC7143997

[27] Trujillo EB, Claghorn K, Dixon SW, Hill EB, Braun A, Lipinski E, et al. Inadequate nutrition coverage in outpatient cancer centers: Results of a national survey. Journal of Oncology. 2019;2019:7462940. doi: 10.1155/2019/7462940 31885583 PMC6893237

[28] Forbes CC, Swan F, Greenley SL, Lind M, Johnson MJ. Physical activity and nutrition interventions for older adults with cancer: a systematic review. Journal of Cancer Survivorship. 2020:1–23. doi: 10.1007/s11764-020-00883-x 32328828 PMC7473955

[29] Ward-Ritacco CL, Adrian AL, Johnson MA, Rogers LQ, Evans EM. Adiposity, physical activity, and muscle quality are independently related to physical function performance in middle-aged postmenopausal women. Menopause (New York, NY). 2014;21(10):1114–21. doi: 10.1097/GME.0000000000000225 24618768

[30] Mikkelsen MK, Lund CM, Vinther A, Tolver A, Johansen JS, Chen I, et al. Effects of a 12-week multimodal exercise intervention among older patients with advanced cancer: Results from a randomized controlled trial. The Oncologist. 2022;27(1):67–78. doi: 10.1002/onco.13970 34498352 PMC8842365

[31] Morton RW, Murphy KT, McKellar SR, Schoenfeld BJ, Henselmans M, Helms E, et al. A systematic review, meta-analysis and meta-regression of the effect of protein supplementation on resistance training-induced gains in muscle mass and strength in healthy adults. British Journal of Sports Medicine. 2018;52(6):376–84. doi: 10.1136/bjsports-2017-097608 28698222 PMC5867436

[32] Cermak NM, Res PT, de Groot LC, Saris WH, van Loon LJ. Protein supplementation augments the adaptive response of skeletal muscle to resistance-type exercise training: a meta-analysis. The American journal of clinical nutrition. 2012;96(6):1454–64. doi: 10.3945/ajcn.112.037556 23134885

[33] Finger D, Goltz FR, Umpierre D, Meyer E, Rosa LH, Schneider CD. Effects of protein supplementation in older adults undergoing resistance training: a systematic review and meta-analysis. Sports Med. 2015;45(2):245–55. doi: 10.1007/s40279-014-0269-4 25355074

[34] Liao C-D, Lee P-H, Hsiao D-J, Huang S-W, Tsauo J-Y, Chen H-C, et al. Effects of Protein Supplementation Combined with Exercise Intervention on Frailty Indices, Body Composition, and Physical Function in Frail Older Adults. Nutrients. 2018;10(12):1916. doi: 10.3390/nu10121916 30518122 PMC6315527

[35] Gielen E, Beckwée D, Delaere A, De Breucker S, Vandewoude M, Bautmans I. Nutritional interventions to improve muscle mass, muscle strength, and physical performance in older people: an umbrella review of systematic reviews and meta-analyses. Nutrition reviews. 2021;79(2):121–47. doi: 10.1093/nutrit/nuaa011 32483625

[36] Pedersen AN, Kondrup J, Børsheim E. Health effects of protein intake in healthy adults: a systematic literature review. Food & Nutrition Research. 2013;57(1):21245. doi: 10.3402/fnr.v57i0.21245 23908602 PMC3730112

[37] Orsso CE, Montes-Ibarra M, Findlay M, van der Meij BS, de van der Schueren MAE, Landi F, et al. Mapping ongoing nutrition intervention trials in muscle, sarcopenia, and cachexia: a scoping review of future research. Journal of Cachexia, Sarcopenia and Muscle.n/a(n/a). doi: 10.1002/jcsm.12954 35301816 PMC9178172

[38] Presley CJ, Dotan E, Soto-Perez-de-Celis E, Jatoi A, Mohile SG, Won E, et al. Gaps in nutritional research among older adults with cancer. Journal of Geriatric Oncology. 2016;7(4):281–92. doi: 10.1016/j.jgo.2016.04.006 27197919 PMC4969118

[39] Santagnello SB, Martins FM, de Oliveira Junior GN, de Freitas Rodrigues de Sousa J, Nomelini RS, Murta EFC, et al. Improvements in muscle strength, power, and size and self-reported fatigue as mediators of the effect of resistance exercise on physical performance breast cancer survivor women: a randomized controlled trial. Supportive Care in Cancer. 2020:1–10. doi: 10.1007/s00520-020-05429-6 32306100

[40] Madzima TA, Ormsbee MJ, Schleicher EA, Moffatt RJ, Panton LB. Effects of resistance training and protein supplementation in breast cancer survivors. Medicine & Science in Sports & Exercise. 2017;49(7):1283–92. doi: 10.1249/MSS.0000000000001250 28252552

[41] van Dongen EJI, Haveman-Nies A, Doets EL, Dorhout BG, de Groot LCPGM. Effectiveness of a diet and resistance exercise intervention on muscle health in older adults: ProMuscle in practice. Journal of the American Medical Directors Association. 2020;21(8):1065–72.e3. 31948853 10.1016/j.jamda.2019.11.026

[42] Unterberger S, Aschauer R, Zöhrer PA, Draxler A, Franzke B, Strasser E-M, et al. Effects of an increased habitual dietary protein intake followed by resistance training on fitness, muscle quality and body composition of seniors: A randomised controlled trial. Clinical Nutrition. 2022;41(5):1034–45. doi: 10.1016/j.clnu.2022.02.017 35390727

[43] Houben LH, Overkamp M, Van Kraaij P, Trommelen J, Van Roermund JG, De Vries P, et al. Resistance exercise training increases muscle mass and strength in prostate cancer patients on androgen deprivation therapy. Medicine and science in sports and exercise. 2023;55(4):614. doi: 10.1249/MSS.0000000000003095 36534950 PMC9997646

[44] Hunter GR, McCarthy JP, Bamman MM. Effects of resistance training on older adults. Sports Medicine. 2004;34(5):329–48. doi: 10.2165/00007256-200434050-00005 15107011

[45] Strasser B, Steindorf K, Wiskemann J, Ulrich CM. Impact of resistance training in cancer survivors: a meta-analysis. Medicine & Science in Sports & Exercise. 2013;45(11):2080–90. doi: 10.1249/MSS.0b013e31829a3b63 23669878

[46] Winters-Stone KM, Dobek J, Bennett JA, Nail LM, Leo MC, Schwartz A. The effect of resistance training on muscle strength and physical function in older, postmenopausal breast cancer survivors: a randomized controlled trial. Journal of Cancer Survivorship. 2012;6(2):189–99. doi: 10.1007/s11764-011-0210-x 22193780 PMC3488606

[47] Serra MC, Ryan AS, Ortmeyer HK, Addison O, Goldberg AP. Resistance training reduces inflammation and fatigue and improves physical function in older breast cancer survivors. Menopause (New York, NY). 2018;25(2):211. doi: 10.1097/GME.0000000000000969 28832427 PMC5771834

[48] Winters-Stone KM, Torgrimson-Ojerio B, Dieckmann NF, Stoyles S, Mitri Z, Luoh S-W. A randomized-controlled trial comparing supervised aerobic training to resistance training followed by unsupervised exercise on physical functioning in older breast cancer survivors. Journal of Geriatric Oncology. 2022;13(2):152–60. doi: 10.1016/j.jgo.2021.08.003 34426142 PMC9003120

[49] Winters-Stone KM, Boisvert C, Li F, Lyons KS, Beer TM, Mitri Z, et al. Delivering exercise medicine to cancer survivors: has COVID-19 shifted the landscape for how and who can be reached with supervised group exercise? Supportive Care in Cancer. 2022;30(3):1903–6. doi: 10.1007/s00520-021-06669-w 34741653 PMC8571667

[50] Hill JR, Harrington AB, Adeoye P, Campbell NL, Holden RJ. Going Remote—Demonstration and Evaluation of Remote Technology Delivery and Usability Assessment With Older Adults: Survey Study. JMIR mHealth and uHealth. 2021;9(3):e26702. doi: 10.2196/26702 33606655 PMC7935399

[51] Dickinson A, Arnott J, Prior S. Methods for human–computer interaction research with older people. Behaviour & Information Technology. 2007;26(4):343–52.

[52] Franz RL, Munteanu C, Neves BB, Baecker R, editors. Time to retire old methodologies? Reflecting on conducting usability evaluations with older adults. Proceedings of the 17th International Conference on Human-Computer Interaction with Mobile Devices and Services Adjunct; 2015.

[53] Frydman JL, Gelfman LP, Goldstein NE, Kelley AS, Ankuda CK. The Digital Divide: Do Older Adults with Serious Illness Access Telemedicine? Journal of General Internal Medicine. 2021. doi: 10.1007/s11606-021-06629-4 33559064 PMC7870026

[54] Furness K, Sarkies MN, Huggins CE, Croagh D, Haines TP. Impact of the method of delivering electronic health behavior change interventions in survivors of cancer on engagement, health behaviors, and health outcomes: Systematic review and meta-analysis. Journal of Medical Internet Research. 2020;22(6):e16112. doi: 10.2196/16112 32574147 PMC7381039

[55] Chan JM, Van Blarigan EL, Langlais CS, Zhao S, Ramsdill JW, Daniel K, et al. Feasibility and Acceptability of a Remotely Delivered, Web-Based Behavioral Intervention for Men With Prostate Cancer: Four-Arm Randomized Controlled Pilot Trial. J Med Internet Res. 2020;22(12):e19238. doi: 10.2196/19238 33382378 PMC7808895

[56] Allicock M, Kendzor D, Sedory A, Gabriel KP, Swartz MD, Thomas P, et al. A pilot and feasibility mobile health intervention to support healthy behaviors in african american breast cancer survivors. Journal of Racial and Ethnic Health Disparities. 2021;8(1):157–65. doi: 10.1007/s40615-020-00767-x 32385847

[57] Blair CK, Harding E, Wiggins C, Kang H, Schwartz M, Tarnower A, et al. A Home-Based Mobile Health Intervention to Replace Sedentary Time With Light Physical Activity in Older Cancer Survivors: Randomized Controlled Pilot Trial. JMIR Cancer. 2021;7(2):e18819. doi: 10.2196/18819 33847588 PMC8087341

[58] Gorzelitz JS, Stoller S, Costanzo E, Gangnon R, Koltyn K, Dietz AT, et al. Improvements in strength and agility measures of functional fitness following a telehealth-delivered home-based exercise intervention in endometrial cancer survivors. Supportive Care in Cancer. 2022;30(1):447–55. doi: 10.1007/s00520-021-06415-2 34304292 PMC9362897

[59] Butcher NJ, Monsour A, Mew EJ, Chan A-W, Moher D, Mayo-Wilson E, et al. Guidelines for Reporting Outcomes in Trial Protocols: The SPIRIT-Outcomes 2022 Extension. JAMA. 2022;328(23):2345–56. doi: 10.1001/jama.2022.21243 36512367

[60] Guralnik JM, Simonsick EM, Ferrucci L, Glynn RJ, Berkman LF, Blazer DG, et al. A short physical performance battery assessing lower extremity function: association with self-reported disability and prediction of mortality and nursing home admission. Journal of gerontology. 1994;49(2):M85–M94. doi: 10.1093/geronj/49.2.m85 8126356

[61] Hays RD, Sherbourne CD, Mazel RM. The RAND 36‐item health survey 1.0. Health economics. 1993;2(3):217–27. doi: 10.1002/hec.4730020305 8275167

[62] Cases MG, Frugé AD, Jennifer F, Locher JL, Cantor AB, Smith KP, et al. Detailed methods of two home-based vegetable gardening intervention trials to improve diet, physical activity, and quality of life in two different populations of cancer survivors. Contemporary clinical trials. 2016;50:201–12. doi: 10.1016/j.cct.2016.08.014 27565830 PMC5055381

[63] Bandura A. Social cognitive theory: An agentic perspective. Annual Review of Psychology. 2001;52(1):1–26. doi: 10.1146/annurev.psych.52.1.1 11148297

[64] Janz NK, Becker MH. The health belief model: A decade later. Health Education Quarterly. 1984;11(1):1–47. doi: 10.1177/109019818401100101 6392204

[65] Rock CL, Thomson CA, Sullivan KR, Howe CL, Kushi LH, Caan BJ, et al. American Cancer Society nutrition and physical activity guideline for cancer survivors. CA: A Cancer Journal for Clinicians. 2022. doi: 10.3322/caac.21719 35294043

[66] US Department of Agriculture and US Department of Health and Human Services. Dietary Guidelines for Americans, 2020–2025. Washington, DC: US Department of Agriculture and US Department of Health and Human Services; 2020.

[67] American College of Sports Medicine. American College of Sports Medicine position stand. Progression models in resistance training for healthy adults. Medicine and science in sports and exercise. 2009;41(3):687–708. doi: 10.1249/MSS.0b013e3181915670 19204579

[68] Schmitz KH, Courneya KS, Matthews C, Demark-Wahnefried W, Galvão DA, Pinto BM, et al. American college of sports medicine roundtable on exercise guidelines for cancer survivors. Medicine & Science in Sports & Exercise. 2010;42(7):1409–26. doi: 10.1249/MSS.0b013e3181e0c112 20559064

[69] Chodzko-Zajko WJ, Proctor DN, Singh MAF, Minson CT, Nigg CR, Salem GJ, et al. Exercise and physical activity for older adults. Medicine & science in sports & exercise. 2009;41(7):1510–30. 19516148 10.1249/MSS.0b013e3181a0c95c

[70] Focht BC, Rejeski WJ, Hackshaw K, Ambrosius WT, Groessl E, Chaplow ZL, et al. The Collaborative Lifestyle Intervention Program in Knee Osteoarthritis Patients (CLIP-OA) trial: Design and methods. Contemporary clinical trials. 2022;115:106730. doi: 10.1016/j.cct.2022.106730 35283261 PMC9426348

[71] Focht BC, Lucas AR, Grainger E, Simpson C, Fairman CM, Thomas-Ahner JM, et al. Effects of a group-mediated exercise and dietary intervention in the treatment of prostate cancer patients undergoing androgen deprivation therapy: Results From the IDEA-P trial. Annals of Behavioral Medicine. 2018;52(5):412–28. doi: 10.1093/abm/kax002 29684136 PMC6361261

[72] Schulz KF, Altman DG, Moher D. CONSORT 2010 statement: updated guidelines for reporting parallel group randomised trials. Trials. 2010;11(1):1–8. 21350618 10.4103/0976-500X.72352 PMC3043330

[73] National Cancer Institute. Diet History Questionnaire III. Washington, DC: U.S. Department of Health and Human Services; 2018.

[74] United States Department of Agriculture. Dietary Guidelines for Americans, 2020–2025. US Department of Agriculture and US Department of Health and Human Services; 2020.

[75] Amireault S, Godin G, Lacombe J, Sabiston CM. The use of the Godin-Shephard Leisure-Time Physical Activity Questionnaire in oncology research: a systematic review. BMC medical research methodology. 2015;15(1):1–11. doi: 10.1186/s12874-015-0045-7 26264621 PMC4542103

[76] Tan S, Turner J, Kerin-Ayres K, Butler S, Deguchi C, Khatri S, et al. Health concerns of cancer survivors after primary anti-cancer treatment. Supportive Care in Cancer. 2019;27(10):3739–47. doi: 10.1007/s00520-019-04664-w 30710242

[77] Calogiuri G, Nordtug H, Weydahl A. The potential of using exercise in nature as an intervention to enhance exercise behavior: Results from a pilot study. Perceptual and motor skills. 2015;121(2):350–70. doi: 10.2466/06.PMS.121c17x0 26348226

[78] Ware JE Jr., Bayliss MS, Rogers WH, Kosinski M, Tarlov AR. Differences in 4-year health outcomes for elderly and poor, chronically ill patients treated in HMO and fee-for-service systems. Results from the Medical Outcomes Study. JAMA. 1996;276(13):1039–47. 8847764

[79] Ware JE Jr, Sherbourne CD. The MOS 36-item short-form health survey (SF-36): I. Conceptual framework and item selection. Medical Care. 1992:473–83. 1593914

[80] Umstattd MR, Motl R, Wilcox S, Saunders R, Watford M. Measuring physical activity self-regulation strategies in older adults. Journal of Physical Activity and Health. 2009;6(s1):S105–S12. doi: 10.1123/jpah.6.s1.s105 19998856

[81] Norman CD, Skinner HA. eHEALS: The eHealth Literacy Scale. J Med Internet Res. 2006;8(4):e27. doi: 10.2196/jmir.8.4.e27 17213046 PMC1794004

[82] Harris PA, Taylor R, Thielke R, Payne J, Gonzalez N, Conde JG. Research electronic data capture (REDCap)—a metadata-driven methodology and workflow process for providing translational research informatics support. Journal of biomedical informatics. 2009;42(2):377–81. doi: 10.1016/j.jbi.2008.08.010 18929686 PMC2700030

