# Supplementary material for: E-PROOF: E-intervention for protein intake and resistance training to optimize function: A study protocol
Source: PLoS One. 2024 May 8;19(5):e0302727. doi: 10.1371/journal.pone.0302727 (PMC11078354; doi:10.1371/journal.pone.0302727)
Supplement: S1 File — (DOCX) [file pone.0302727.s001.docx]

*PROTOCOL*

TITLE: E-PROOF: E-intervention for Protein Intake and Resistance Training to Optimize Function

PI: Jessica Krok-Schoen, PhD, Assistant Professor, School of Health and Rehabilitation Sciences, College of Medicine, The Ohio State University

TYPE OF STUDY: Behavioral Intervention

**BACKGROUND**

The 12 million older (age ≥65) cancer survivors (OCS) are at heightened risk of physical function impairment, one of the most pervasive health challenges at the intersection of aging and cancer.[^1^](#_ENREF_1) Impairments in physical function are more prevalent among older adults with a history of cancer (24-58%) compared to the general older adult population (20-32%), with increased incidence with age.[^2-6^](#_ENREF_2) Compared to non-cancer peers, older adults with a history of cancer are also more likely to become fatigued faster and have comorbidities, including higher rates of heart and lung disease, arthritis, pain, and obesity.[^2^](#_ENREF_2)^,^[^5^](#_ENREF_5)^,^[^7-10^](#_ENREF_7) This information underscores the need to design effective interventions to prevent or reduce physical function impairment,[^2^](#_ENREF_2)^,^[^11^](#_ENREF_11) which has multiple deleterious health effects, including poor health-related quality of life (HRQoL) and higher all-cause mortality.[^12-14^](#_ENREF_12) OCS are at particular risk of physical function impairment compared to both younger cancer patients and the general older adult population.[^5^](#_ENREF_5)^,^[^6^](#_ENREF_6)^,^[^15^](#_ENREF_15) Despite the clinical importance and rising prevalence of physical function impairment, physical function has not always been thoroughly examined among OCS. *Thus, there is a critical knowledge gap about how physical function* *can be improved among OCS.* Consequences of not addressing this serious need will be reduced HRQoL, and increased healthcare costs among the growing OCS population.[^16^](#_ENREF_16)^,^[^17^](#_ENREF_17)

Declines in adequate protein and calorie intake results in accelerated loss of muscle mass and physical functioning in older adults.[^18-20^](#_ENREF_18) Prior randomized trials demonstrate that increased protein intake coupled with resistance training, is the most effective strategy to improve physical function.[^19^](#_ENREF_19)^,^[^21^](#_ENREF_21)^,^[^8^](#_ENREF_8)^,^[^22-25^](#_ENREF_22) Despite substantial evidence, there is a paucity of research on protein consumption and resistance training among OCS, a population at high risk for functional limitations. Given the ongoing COVID-19 pandemic and the positive impact of online behavior change interventions for cancer survivors,[^26-34^](#_ENREF_26) online-based interventions to improve physical function should be tested among OCS. A novel approach to improving physical function in OCS is to utilize online, tailored education and counseling from registered dietitians and exercise scientists, to improve dietary intake (protein intake, diet quality) and participation in resistance exercise. The proposed study will be the first synchronous, online protein-focused diet and resistance training intervention among OCS.

**SPECIFIC AIMS**

Our objective is to improve physical function through online, tailored nutritional and resistance training counseling to increase protein intake, improve diet quality, increase resistance training, and improve health outcomes (muscle strength, dietary quality, HRQoL, self-efficacy, weight management). This online delivery mechanism also decreases participant exposure to COVID-19 among susceptible OCS. We will utilize established strategies to improve physical function in an innovative way through a 12-week synchronous, online counseling and education intervention for OCS at high risk for physical function impairment. Recruitment will take place at The Ohio State University Comprehensive Cancer Center. Our rationale is that understanding the influence of nutritional and resistance training counseling and education among OCS is likely to advance the science of geriatrics and cancer survivorship. This study, the E-PROOF (**E**-intervention for **P**rotein Intake and **R**esistance Training t**o** **O**ptimize **F**unction) study, builds on our team’s expertise in geriatrics, cancer survivorship, diet, exercise, and physical function and our preliminary data on the dietary, exercise, and physical function deficits among OCS. To attain the overall objective, two aims will be pursued:

**Aim 1:** Determine the feasibility and acceptability of implementing a 12-week online dietary and resistance training randomized trial with 70 older cancer survivors. This aim will be accomplished by collecting detailed process data, which allows the assessment of accrual, retention, and adherence. Our *hypothesis* is that we will recruit (within 9 months) 70 OCS, retain ≥80% of the sample, and maintain ≥80% adherence to the intervention.

**Aim 2:** Examine the preliminary efficacy of this 12-week online dietary and resistance training intervention with the intervention participants. Registered dietitians and exercise scientists will provide these participants with tailored dietary and resistance training recommendations, counseling, and educational materials. Our *hypothesis* is that intervention participants will experience statistically significant, clinically meaningful improvements in physical function and associated health outcomes (i.e., muscle strength, dietary quality, HRQoL, self-efficacy, weight management) after the 12-week dietary and resistance training intervention and at a 3-month post-intervention follow-up.

**METHODS**

1. **STUDY DESIGN**

We will examine the feasibility and preliminary efficacy of a 12-week online dietary and resistance training intervention to improve the physical function of OCS through increased protein consumption, healthy eating, and resistance training. In this pilot randomized controlled trial, participants (n=70) will be randomized to one of the two groups: experimental (online counseling, menus, educational materials) and enhanced control (passive educational materials). We will examine the intervention effects on physical function and associated health outcomes during the 12-week period between the experimental and control group.

1. **PARTICIPANT SELECTION**
   1. *ELIGIBILITY CRITERIA*

Eligibility criteria are: ≥65 years of age, a primary diagnosis of stage I-III breast, colorectal, and prostate cancer, completion of primary curative treatment, reported at least 1 physical function limitation on the PFSS (“limited a little”, “limited a lot”), no evidence of progressive disease or second cancers, community-dwelling, and able to provide consent. The 3 cancer types are chosen because they are the most common cancer types[^35^](#_ENREF_35) with high 5-year survival rates.[^36^](#_ENREF_36) Additionally, most of the evidence of the benefits of exercise and diet has occurred in survivors diagnosed with breast, prostate, or colorectal cancer.[^24^](#_ENREF_24)^,^[^37-39^](#_ENREF_37) Individuals are excluded if they currently receive cancer treatment (e.g., chemotherapy, radiation), have liver and/or renal disease limiting their protein intake, are under the care of a RD/nutritionist, participating in other diet/exercise interventions, consume protein supplements, and have contraindications to unsupervised exercise (e.g., walker/wheelchair use).

- 1. *RECRUITMENT*

Four methods will be employed to recruit potential participants:

1. Flyers distributed at the JamesCare for Life programs (wellness programs offered through the Ohio State University Comprehensive Cancer Center (OSUCCC)) as well as through their email list of cancer survivors. Flyers will also be distributed at the OSUCCC’s Cancer and Aging Resiliency (CARE) clinics, multidisciplinary clinics designed to address the unique needs of older cancer survivors, and the OSUCCC GI Medical Oncology Clinic The study will be advertised on the flyer and eligible women and men will be requested to contact the research assistant if they are interested in study participation.

2. In-person during follow-up visits at the Survivorship Clinic and CARE clinics within OSUCCC by study team member (Dr. Rosko). When prospective patients are identified, the provider will provide the prospective participant with the recruitment flyer that has the PI and research assistant’s names and contact information. In addition, the research assistant will be in the clinics screening eligible participants by appointment date and meeting the potential participants in person to recruit them.

3. Previously consented older cancer survivors in the OSUCCC Total Cancer Care (TCC) registry. The TCC registry, a personalized cancer care initiative designed to collect clinical data and tumor specimens throughout a patient's lifetime, will be utilized to capture a larger cancer populations. TCC’s consent rate is 94% of all patients evaluated at the OSUCCC. Based on the eligibility criteria, TCC will contact potential participants, identified across all OSUCCC clinics, through phone calls, to introduce the proposed study and see if they agree to be contacted by the study staff. If permission is given, the study staff member will contact the potential participant and determine eligibility. The study staff member will administer the 10-question RAND-36 Physical Function Subscale (PFSS) to determine physical function limitations. If the participant has at least 1 limitation on the PFSS, the participant will be invited to participate in the study, consented, and invited to the baseline in-person visit. These recruitment procedures will continue for 9 months until 70 older cancer survivors have agreed to participate in the study. Previous efforts by the PI, co-Is, and consultants to recruit older cancer survivors have shown these strategies to be effective.

4. Facebook and Research Match Recruitment Messages. OSU Center for Clinical and Translational Science (CCTS) services will be used to generate advertisements for Facebook and Research Match that align with The Ohio State University’s College of Medicine institutional branding standards. Facebook advertisements will use the following criteria as the target audience: ≥65 years old, geographic location within a 90-minute driving radius of Columbus, Ohio. The Facebook advertisement will direct the user to the E-PROOF Study Search page. These advertisements will be live for a two-week period during recruitment.

Research Match will also be used to recruit potential participants. ResearchMatch is a free and secure online tool created by academic institutions across the country to share study and PI contact information to potentially eligible participants. Potentially eligible participants will receive an email introduction to the study and, if they are interested, permit ResearchMatch to release their contact information to the study team. The research assistant will then contact potential participants, via email or phone, to explain study requirements, and confirm eligibility (≥65 years of age, history of stage 1-3 breast, colorectal, and prostate cancer, completed primary cancer treatment, and have an email address).

B. Recruitment Locations

Recruitment will occur at the Survivorship Clinic and CARE clinics within OSUCCC. Recruitment will occur in the clinic exam rooms and/or the waiting room at these clinics. As noted in the facilities page, OSUCCC is the third largest cancer hospital in the nation. The hospital includes a 36-bed blood and marrow transplant unit, inpatient units in which sub-specialists focus on just one type of cancer, a cancer clinical trials unit, 14 state-of-the-art operating rooms, six interventional radiology suites, special isolation rooms, chemotherapy and transfusion areas, and seven linear accelerators for radiation therapy. The Survivorship Clinic at OSUCCC is one of the country’s largest cancer supportive care programs, with about 40 team members. The Cancer and Aging Resiliency (CARE) clinics at OSUCCC provides each older adult with cancer with a comprehensive, multidisciplinary treatment plan. Study team member, Dr. Rosko, is the director of the CARE clinics located at the OSUCCC.

C. Recruitment Follow-up

The research assistant will send out a reminder mailing to all identified participants two weeks after the initial study contact. The research assistant will make at three attempts by phone to reach the non-responders. If no response is given after the third attempt, the potential participant will be considered a “non-responder.”

- 1. *INFORMED CONSENT PROCESS*

For in-person informed consent, it will take place in a quiet setting and in a manner such that the participant does not feel rushed or pressured. If telephone/video-based informed consent is received, the potential participants will be mailed two paper copies of the informed consent (one to send back to study team and one to keep for their records) and asked to review them prior to the telephone call with study staff.

In both in-person and telephone/video-based informed consent procedures, subjects will be informed about all aspects of the study and asked to read the consent form. The form will be written in simple, easy-to-understand language. We require study staff to review all key aspects of the study verbally. Staff will be provided with a structured checklist for this purpose. As the participant reads each page, they are asked to initial the page and encouraged to ask questions. Prior to signing the document, they will be asked basic questions about the study to ascertain as best as possible if they have understood the requirements of the study, the potential risks, and the potential benefits. A copy of the informed consent will be given to the participant, and the original signed copies will be kept in the participant’s file.

- 1. *RISKS AND BENEFITS*

All data obtained from participants is considered confidential. Precautions will be taken to protect computerized or electronic data from unauthorized disclosure, tampering, or damage by controlling access to the computers and files that hold this information. Access to computer files will only be obtained through passwords and will only be accessed by IRB-authorized research study personnel. Only authorized research study personnel will have permission to examine computer records to identify which patients are eligible to approach. Analysis of data will be performed at The Ohio State University by the study PI and with assistance from Dr. Xu (OSU College of Medicine). Whereas no assurance can be made to an individual participant that he or she will personally benefit from such research, the experience should be beneficial. The risks compared to the potential benefits are minimal to the individual research participant and virtually nonexistent to others or society in general. More information about the risks and benefits are on pages 8-10.

1. **STUDY PROCEDURES**
   1. *WHAT WILL BE DONE*

The study’s overall goal is to recruit 70 older cancer survivors to examine the feasibility and preliminary efficacy of a 12-week online dietary and resistance training intervention to improve their physical function through increased protein consumption, healthy eating, and resistance training.

- 1. *HOW IT WILL BE DONE*

The E-PROOF intervention core content is from publicly available, pre-existing educational content provided by the NIA, NCI, American Cancer Society, American College of Sports Medicine (ACSM), and OSUCCC. The dietary core content includes: 1) recommended amount and sources of protein intake; 2) healthy eating as an OCS; 3) protein intake benefits; and 4) importance of maintaining physical function through diet. The resistance training intervention core content includes: 1) recommended frequency and resistance training exercises; 2) resistance training benefits; 3) different muscle groups; and 4) the importance of maintaining physical function through resistance training. Intervention participants will also have the option of attending Zoom^®^-based social hours (held at 7-8pm EST, 1 day/week, weeks 3, 6, 9) to share their thoughts and experiences with each other. Informed design was used for intervention development by reviewing relevant evidence; replicating prior successful intervention elements from the PI and co-Is and; incorporating feedback from the Cancer and Aging Research Group’s patient advocacy group (see Letter of Support).

*Randomization and Baseline Assessment:* Subjects will be block randomized by the number of physical function limitations measured by the PFSS.[^40^](#_ENREF_40) At the in-person visits at baseline and end-of-study, study staff will measure the physical function (SPPB), muscle strength (handgrip, SPPB), height, weight, and waist circumference of all participants (intervention and control). The Research Electronic Data Capture (REDCap) secure platform[^41^](#_ENREF_41)^,^[^42^](#_ENREF_42) will be used for baseline questionnaire data collection, which will assess dietary quality (DHQIII), self-reported exercise, HRQoL, self-efficacy, and demographic and clinical information. After data collection, the research assistant will show intervention participants how to access the REDCap-based logs and enter their weekly dietary intake and exercise adherence. At the end of the baseline visit, intervention participants will receive a binder with printed educational materials on increasing dietary protein, healthy eating, and resistance training to preserve or rebuild lean body mass as a reference tool. Resistance bands (three levels of Thera-bands®), and resistance band exercise guidelines (exercises by muscle group, proper form, how to rate difficulty) will be distributed to intervention participants.

*Intervention Participants*. Participants randomized to the intervention group (n=35) will be paired after the baseline assessment with a RD and ES for the duration of the 12-week intervention **(Figure 1)**. The diet and resistance training intervention will be tailored to each participant in the intervention group based on the information collected during their baseline visit.

**Figure 1. Study schema**


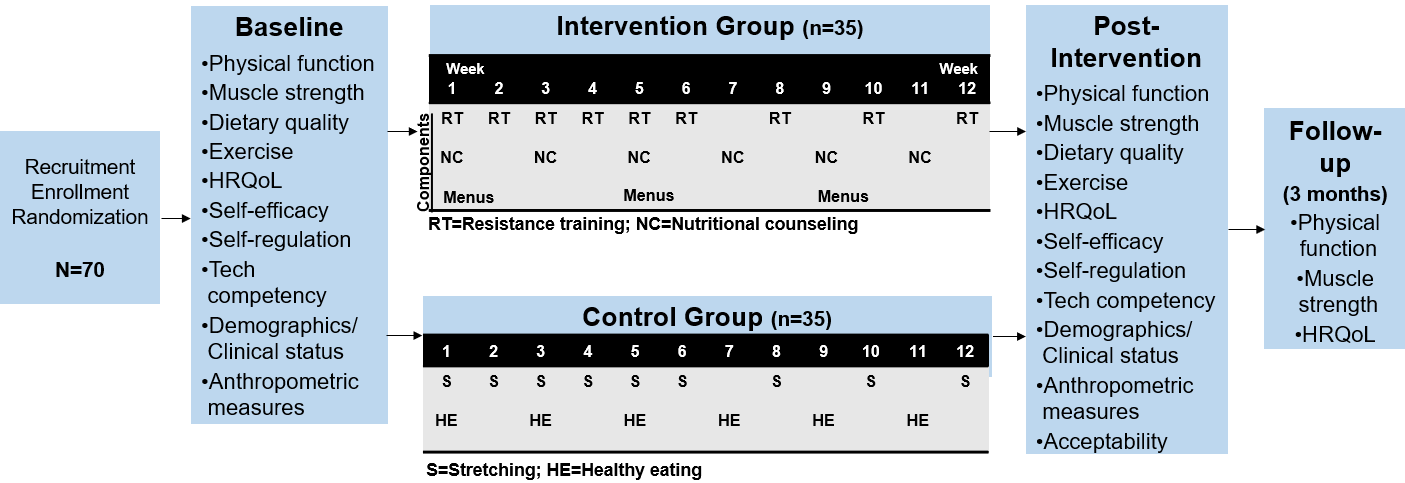


Nutritional counseling: Within 1 week of the baseline visit, a RD will review the DHQIII results and create a tailored dietary plan focusing on protein intake and healthy eating for each intervention participant. At weeks 1, 3, 5, 7, 9, and 11, the RD will provide one-on-one, online (Zoom^®^, FaceTime^®^) nutritional counseling to the intervention participants. During the biweekly online 30-minute visits, the RD, guided by elements in behavior change theories, will review and check on their progress toward increased protein intake and healthy eating, use motivational interviewing strategies to support the participants’ progress, provide reinforcement, explore strategies to overcome barriers, field questions, and establish tailored goals. Counseling will consist of education to stimulate sufficient protein- and energy intake using regular food, based on the current American Cancer Society nutrition guidelines for cancer survivors[^43^](#_ENREF_43) and DGAs for protein and energy for adults aged ≥60 (5-6.5 oz. eq/day based on 1,600-2,600 calories/day).[^44^](#_ENREF_44) Counseling of these protein targets will account for variability in body weight.[^45-47^](#_ENREF_45) Due to physiological limit of protein synthetic capacity, spread feeding of proteins over the day is preferred and participants will be advised to consume 25-30 grams of proteins per meal.[^18^](#_ENREF_18) After the counseling session for weeks 1, 5, and 9, the RD will email/mail participants menus, snack ideas, and recipes focused on protein and healthy eating. Participants will be encouraged to record their weekly dietary intake in their REDCap-based log to practice self-monitoring, provide motivation, and facilitate recall and reflection during nutritional counseling sessions. RDs will access these online entries to review participants’ protein and overall dietary intake and provide recommendations during counseling.

Resistance training counseling: For the first 6 weeks, the ES will host weekly, one-on-one, 30-minute, online resistance training sessions for the intervention group, using a progression model.[^48^](#_ENREF_48) After week 6, the resistance training sessions will taper, and be held biweekly at weeks 8, 10, and 12. Based on behavior change models, at the beginning of all sessions, the ES will review progress toward increased resistance training, and support progress and strategies to overcome barriers through motivational interviewing. In each training session, the ES will provide education, then lead participants through a 5-minute warm-up, followed by a series of whole-body exercises (each major muscle group) performed at each session, and a 5-minute cool-down. The training is based on a co-I’s (Focht) expertise in exercise interventions,[^49-51^](#_ENREF_49) the ACSM exercise guidelines for cancer survivors,[^52^](#_ENREF_52) and ACSM recommendations for progressive resistance training for older adults[^53^](#_ENREF_53) consisting of 1-3 sets of 8-10 exercises with 8-12 repetitions with a 1-2 min rest between sets. Participants will be encouraged to complete these exercises two more times/week, totaling three sessions/week, and record their weekly resistance trainings in the REDCap platform (same weekly log as dietary intake). The diary will ask participants if they exercised (yes/no), type of exercise (i.e., resistance training, walking), intensity (mild/moderate/strenuous) and the time spent exercising for each day of the week. Diaries will be used by the ES to assess exercise adherence and to progress the exercise program during training sessions. Participants will be encouraged to refer to their educational materials for guidance between synchronous resistance training sessions.

*Enhanced Control Participants.* Participants randomized to the enhanced control group (n=35) will not receive online nutritional or resistance training education or counseling. Control participants will take part in three in-person assessments (baseline, end-of-study, 3-month follow-up) with study staff and receive printed materials (NCI’s “Facing Forward: Life After Cancer Treatment”) pertaining to general cancer survivorship.

*Follow-up*. Three months after the 12-week intervention, all participants will return for an in-person assessment by study staff of physical function, muscle strength, and HRQoL. This information is essential to understanding the sustained impact of this intervention and trajectories of physical function among OCS.

- 1. *TIMELINE*

We will work with cancer clinic leadership, clinicians, and clinic staff to coordinate recruitment to ensure that clinic flow is not disrupted. The Clinical Research Coordinator, TBN, will serve as a central point of contact and will assist with these activities. Throughout the course of the study period, Dr. Krok-Schoen will hold regular staff meetings to discuss recruitment, and to address any issues that occur on a case-by-case and clinic-by-clinic basis. The Clinical Research Coordinator will directly supervise the day-to-day progress of all study activities. We estimate a large number (n~150) of potentially eligible patients on an average weekday at the OSUCCC, with a limiting factors being workforce, clinic flow, and participation rates. We anticipate that we can feasibly enroll an average of 2 patients per day over 9 months of recruitment and reach our recruitment goal of 70 participants.

**Figure 2. Timeline and Work Plan**

|  | **Year 1** | | | | **Year 2** | | | |
| --- | --- | --- | --- | --- | --- | --- | --- | --- |
| Study Quarters (over 2 years) | **1** | **2** | **3** | **4** | **1** | **2** | **3** | **4** |
| ***Participant Recruitment*** | | | | | | | | |
| Recruitment of 70 older cancer survivors |  |  |  |  |  |  |  |  |
| ***Aim 1: Determine Feasibility and Acceptability of Intervention*** | | | | | | | | |
| Implementation of a 12-week online, tailored nutritional and resistance training counseling intervention |  |  |  |  |  |  |  |  |
| Measurement of accrual, retention, and adherence rates |  |  |  |  |  |  |  |  |
| Analysis of participant intervention evaluation |  |  |  |  |  |  |  |  |
| ***Aim 2: Examine Efficacy of Intervention*** | | | | | | | | |
| Measurement of physical function, muscle strength, and height, weight, and waist circumference at baseline and 12 weeks |  |  |  |  |  |  |  |  |
| Measurement of diet quality, exercise, health-related quality of life, self-efficacy, and demographic and clinical information through REDCap-based questionnaires at baseline and 12 weeks |  |  |  |  |  |  |  |  |
| Analysis of change in physical function, muscle strength, and height, weight, and waist circumference from baseline to 12 weeks |  |  |  |  |  |  |  |  |
| Analysis of change in diet quality, exercise, health-related quality of life, self-efficacy, and demographic and clinical information from baseline to 12 weeks |  |  |  |  |  |  |  |  |
| ***Examine Maintenance of Intervention*** | | | | | | | | |
| Measurement of physical function, muscle strength, and health-related quality of life at 3-month follow-up |  |  |  |  |  |  |  |  |
| Analysis of change baseline and end-of-study (6 months) in physical function, muscle strength, and health-related quality of life |  |  |  |  |  |  |  |  |
| ***Integration and Dissemination Activities*** | | | | | | | | |
| Project meetings, monitoring, and fidelity checks |  |  |  |  |  |  |  |  |
| Prepare abstracts, manuscripts, and presentations |  |  |  |  |  |  |  |  |
| Preparation and submission of next-step grant application |  |  |  |  |  |  |  |  |

**DATA COLLECTION AND MANAGEMENT PROCESS**

- 1. *DATA TO BE COLLECTED*

We are collecting data using all validated measures. Our main outcome is physical function which will be measured by The physical performance assessment, Short Physical Performance Battery (SPPB)[^54^](#_ENREF_54), will measure physical function at baseline, end-of-study, and follow-up. The SPPB includes three lower extremity physical performance measures (standing balance, five consecutive chair rises, 4-meter gait walk at usual pace) to assess lower extremity strength.

Secondary outcomes will be:

- *Acceptability.* At end-of-study, intervention group participants will complete a 5-item, Likert-scale questionnaire, with written comments, regarding their preferences for receiving education and counseling, successes and challenges, program satisfaction, and suggestions for improvement.
- *Feasibility.* Intervention feasibility will be measured by rates of accrual, retention, and adherence at baseline, end-of-study, and 3-month follow-up. Recruitment rate is based on Consolidated Standards of Reporting Trials criteria[^55^](#_ENREF_55) that includes eligible consented individuals and eligible non-consented individuals with non-recruitment reasons documented. Process information (number/duration of video visits) will be recorded.
- *Muscle Strength*. Handgrip strength will be measured at baseline, end-of-study, and follow-up in both hands using a hydraulic grip strength dynamometer (Jamar Model 7498).
- *Dietary Quality*. Protein intake and dietary quality will be assessed at baseline and end-of-study by the National Cancer Institute’s DHQIII.[^56^](#_ENREF_56)
- *Resistance exercise.* Self-reported resistance exercise will be assessed using the revised version of The Godin-Shephard Leisure-Time Exercise Questionnaire (LTEQ) modified to specifically capture self-reported resistance exercise participation.
- *HRQoL.* RAND-36 Health Status Measure[^57^](#_ENREF_57) is comprised of 8 subscales assessing multiple aspects of HRQoL. Physical and mental component summaries are created. All scores range from 0-100, with 100 as the highest.
- *Self-efficacy.* Baseline and end-of-study self-efficacy for diet and resistance training will be measured by: “How sure are you that you could do exercises to make your body stronger for 15 minutes, 3 days a week?” and “How sure are you that you could improve your diet?”
- *Self-regulation.* Baseline and end-of-study self-regulation for diet and exercise will be measured by the 12-item Dietary Self-Regulation Scale[^58^](#_ENREF_58) and the 12-item Exercise Self-Regulation Scale[^59^](#_ENREF_59).
- *Technology competency.* The 8-item eHealth Literacy Scale (eHEALS)[^60^](#_ENREF_60) will measure knowledge, comfort, and perceived skills of engaging in eHealth at baseline and end-of-study. A 5-point Likert scale will be used.
- *Anthropometry.* Body weight, height, and waist circumference will be collected at baseline and end-of-study.
- *Demographic and Clinical Information*. Participants’ age, race, ethnicity, gender, education, income, marital status, insurance status, cancer diagnosis, stage at diagnosis, time since diagnosis, treatments received (e.g., chemotherapy, radiation) and comorbidities will be collected at baseline and updated at end-of-study.
  1. *HOW THE DATA WILL BE COLLECTED OR ACCESSED*

Research materials will come from the following sources: Total Cancer Care registry data from the study site, and the Cancer Supportive Care Clinic, part of OSUCCC will be used for recruitment purposes. . Participants’ physical function (as measured by the SPPB), muscle strength (handgrip, SPPB), weight, height, and waist circumference will be collected at the in-person meetings (baseline and after the 12-week intervention). Online Research Electronic Data Capture (REDCap)-based questionnaires at baseline and end-of-study will collect assessing dietary quality (DHQIII), self-reported exercise (frequency/duration/intensity), health-related quality of life (RAND-36), and self-efficacy. Basic demographic (e.g., age, race, education) and health characteristics (e.g., comorbidities, stage of cancer at diagnosis, treatments received) will be collected through REDCap questionnaires. These questionnaires will be self-administered and a research assistant will be available to address any technological difficulties. Three months after the 12-week intervention, all participants will return for an in-person assessment of physical function (SPPB), muscle strength (handgrip, SPPB), and health-related quality of life (via REDCap questionnaire).

- 1. *DATA COLLECTION TIMELINE*

This is a two-year study. Figure 2, on pages 6 and 7, illustrates the data collection timeline. Briefly, we will recruit 70 participants in 9 months. After recruitment and consent, participants will begin their 12- week intervention. Data will be collected at baseline, end-of-study (12 weeks), and follow-up (3 months after the end of study).

- 1. *HOW THE DATA ARE STORED AND PROTECTED*

The proposed intervention utilizes an individualized approach and does not operate in group settings, therefore ensuring participants’ privacy. Confidentiality will be protected in several ways. All data points will be collated and stored on a REDCap database, a secure web application. Data will be used only in aggregate and no identifying characteristics of individuals will be published or presented. Confidentiality of data will be maintained by using research identification numbers that uniquely identify each individual. Safeguards will be established to ensure the security and privacy of participants' study records. The information collected from participants in this study has a low potential for abuse, since the data do not address sensitive issues. Nevertheless, appropriate measures will be taken to prevent unauthorized use of study information. All computer systems will be password-protected against intrusion. All network-based communications of confidential information will be encrypted. Data other than demographic information do not use names as an identifier. The research number will be used.

All paper files and computer files with the de-identified data will be stored under lock and key at all times. The files matching participants' names and demographic information with research numbers will be kept in a separate room and will be stored in a locked file that uses a different key from that of all other files. Only study personnel will have access to these files, and they will be asked to sign a document that they agree to maintain the confidentiality of the information. After the study is completed, local data will be stored with other completed research studies in a secured storage vault.

- 1. *WILL DATA BE SENT OUTSIDE OF OSU FOR ANALYSIS?*

No.

1. **HUMAN SUBJECTS INFORMATION**
   1. *POTENTIAL RISKS*

An adverse event or experience is defined as any health-related unfavorable or unintended medical occurrence that happens during the process of screening or after randomization. Non-serious adverse events are defined as conditions that may be unpleasant and bothersome to the participant, such as sore muscles, that do not require discontinuing the study intervention or terminating components of the intervention. These do not require reporting. Finally, serious adverse events are defined as events that may be harmful to the participant and/or may be serious enough to warrant either temporary or permanent discontinuation of the study intervention, either because they are intolerable or because they are judged to be potentially harmful. All serious adverse events require immediate reporting and an assessment of the implications for the continuation of the study and/or modification of the consent form.

Unexpected adverse events are defined as events that are not listed above potential events and are not listed in the consent form. Monitoring for unexpected serious adverse events attributable to the intervention is the responsibility of the study project manager who is trained and has experience in monitoring and reporting events. The project manager will inform the PI of any events. Specific reporting and review requirements are defined for unexpected events, so that, if an unexpected event is found to be related to the intervention, the protocol and consent can be modified.

On-site adverse events are defined as events that occur at a research study site. Events that require immediate notification of the study PI (Dr. Krok-Schoen) and senior medical oncologist (Dr. Rosko) and 24-hour notification of the IRB include: (1) deaths, and (2) health events at that result in immediate hospitalization or medical care. These events are also immediately reported to the rest of the study team, the Data and Safety Monitoring Board, and the NIH.

We anticipate that this study will entail minimal physical and psychological risks for study participants. There is a small risk to participants of sustaining an injury while participating in the intervention. However, physical injury is not anticipated and the trained, certified exercise scientist will provide guidance on how to participate in resistance training, safely and effectively. Participant risk may also include, but not anticipated, psychological distress associated with completion of the online questionnaires. If this occurs, study staff will speak to the participant about the difficulties that the questionnaires have caused and will offer a referral to a mental health professional if necessary. Lastly, we are proposing a synchronous online intervention design to reduce psychological distress and assist with the maintenance of health behaviors among participants.

Potential risks for the registered dietitian and exercise scientist will be minimal. Minor risk, but not anticipated, may include psychological distress associated with potentially negative experiences and feedback from participants (i.e., hard to motivate individuals, reported distressing symptoms). These professionals are from an established programs, experienced in providing guidance and counseling online, and will receive training prior to interacting with the study population.

- 1. *PROTECTIONS AGAINST RISK*

Lifestyle interventions that are personalized to individual functional capacity and activity tolerance have been well-established to be safe for older cancer survivors. As a research group, we have the experience and expertise to ensure the safety of participants and minimize aforementioned risk. There will be an educational session for the registered dietitian and exercise scientist on the unique needs and challenges of older cancer survivors prior to the intervention. The content will be taught by members of the research team, gerontologist (Dr. Krok-Schoen), registered dietitian (Dr. Spees), and an exercise scientist (Dr. Focht), will host a comprehensive training session for the registered dietitian and exercise scientist about the unique needs, goals, and contextual factors of older cancer survivors. This training will detail their duties within this intervention including: discussing the core educational elements (provided within the study binders), ensuring the appropriateness of the registered dietitians and exercise scientist’s guidance and counseling, recommendations, informing them of potential reported barriers to improving dietary quality and resistance training, encourage tailored messaging for older cancer survivors, recording process data (e.g., number of visits, adherence to protocol) within the REDCap platform, accessing the REDCap-based exercise/dietary logs of the intervention participants, and ensuring the safety of participants. In addition, Dr. Spees and Dr. Focht, will be the primary contacts for the registered dietitian and exercise scientist, respectively, if any concerns arise about the safety of participants. The registered dietitian and exercise scientist will also be encouraged to communicate any challenges or difficulties to the PI and/or during the regular meetings with the study team.

As in similar prior lifestyle intervention trials conducted by the study team members, we will obtain a release form from each participant’s primary care physician for involvement in the study. We will ask the primary care physicians to complete a form related to the inclusion and exclusion health conditions—yes/no. If an activity-related injury or illness does occur, the PI (Dr. Krok-Schoen), the co-I (Dr. Focht), who is an exercise scientist, and study member (Dr. Rosko), who is a senior oncologist, will be consulted according to the type of symptom reported. In some instances, it may be appropriate to reduce the participant’s resistance training goals. If the injury or symptoms do not resolve after an appropriate period of time, the participant will be referred to her primary care provider for further evaluation. The participant will be encouraged to follow the primary care provider’s instructions.

After the baseline assessment, participants will be provided (on paper and via email) a weblink to access their REDCap-based weekly logs. The research assistant will provide a demonstration on how to access the REDCap-based logs and enter their weekly dietary intake and exercise adherence. Intervention participants will be advised to contact the research assistant in the event of technical difficulties. Weekly REDCap-based logs of the intervention participant’s dietary intake and exercise adherence will offer the registered dietitian and exercise scientist as well as study team insight into their goal progression. These logs will provide an additional method to monitor potential participant difficulties, which the study team will address, therefore, reducing participant risk.

All study members are trained in methods to reduce risks. All study materials and information will be kept on the OSU Medical Centers’ secure computer network that is behind a firewall and password-protected. Specifically, the data will be housed within The Center for Biostatistics at OSU. At the dissemination phase, the data will be shared and housed within OSUWMC Information Technology Department, Biomedical Informatics program, an extensive research-oriented virtual server and storage area network facility. The Biomedical Informatics program also aligns with and leverages the enterprise support model offered by OSUWMC IT including a support desk and a dedicated security and risk assessment team. Any transfer of the data will be behind an internal, password-protected firewall. All applications and processes that use patient information are governed by HIPAA in the course of daily operations. The study will follow all existing procedures for protecting confidentiality and anonymity.

- 1. *BENEFITS*

For the older cancer survivors participating the intervention, they may receive benefit from their participation in terms of improving their physical function (primary study outcome), learning new resistance training techniques, trying new healthy foods and recipes, deepening their personal health and nutrition knowledge, and improving their physical and mental health outcomes. Participation may result in increased knowledge of personal health risks, ways to improve physical function, how to be active safely and effectively as an older cancer survivor, how to overcome barriers to improve dietary quality and resistance training, and increased self-efficacy to work toward their health goals. This knowledge can motivate them to continue these healthy behaviors to support their life as an older adult with a history of cancer. Participants may also gain a better understanding of technology used in this study including Zoom®, FaceTime®, and REDCap. In addition, participants may feel a sense of rapport and support with the registered dietitian and exercise scientist. The participants, registered dietitian, and exercise scientist may experience pride in knowing that they are contributing to research to improve the health and well-being of cancer survivors. The low level of risk associated with this study is overshadowed by the potential benefits. We believe that the aforementioned risks of this study are minimal and reasonable, when compared to: 1) the scientific knowledge to be gained by performing these studies and; 2) the potential benefits to study subjects.

For others outside this research study, results from this study will provide vital information about improving physical function and associated health outcomes among OCS. It will significantly contribute to the understanding of retention, adherence, and efficacy of diet and resistance training interventions among OCS as well as older adults. This study also will provide information about the acceptability and feasibility of technology-based intervention among older cancer survivors, a population who are often excluded from technology-based interventions. Thus, the multiple anticipated benefits to the subjects and others outweigh the minimal risks for participants.

- 1. *STUDY TERMINATION PROCEDURES*

Participants will be informed that they can stop participating at any time with no penalties to them. If participants decide to stop being in the study, their surveys will be deleted from the REDCap system. However, if participants only choose to answer certain questions without explicitly stating a desire to no longer participate, survey data for those answered questions will be stored.

1. **STATISTICAL METHODS**
   1. *SAMPLE SIZE AND JUSTIFICATION*

A pilot randomized controlled trial with 70 OCS (n=35 for intervention arm; n=35 for control arm) is the most efficient design to accomplish the aims of this pilot study.[^61-63^](#_ENREF_61) For this study, the minimum sample size is calculated using G*Power. Specifying alpha level to be 0.05 (one-sided), expected power to be 0.80, and an expected effect size of 0.50 for the primary outcome (physical function measured by SPPB), the sample size required is 27 per group. Effect size estimates are based on recommendations for clinically meaningful differences for the SPPB[^64^](#_ENREF_64)^,^[^65^](#_ENREF_65) and previous pilot randomized controlled trials utilizing the SPBB reporting effect sizes of similar magnitude.[^66^](#_ENREF_66)^,^[^67^](#_ENREF_67) Similar designed pilot randomized controlled trials[^38^](#_ENREF_38)^,^[^68-70^](#_ENREF_68) used comparable sample sizes to demonstrate acceptability and feasibility.

- 1. *DATA ANALYSIS PLAN*

*Aim 1*: The feasibility of implementing a 12-week online diet and resistance training intervention with OCS will be determined by the study accrual rate, retention rate, and adherence rate. The study accrual rate will be calculated by dividing the number of potential participants that passed screening by the total number of participants started in the study after appropriate informed consent procedures. The recruitment goal is set at 70 participants recruited over a 9-month period. Retention rate will be calculated by dividing the total number of participants initiated by total number of participants in the study at baseline and 12 weeks (end-of-study). Retention goal for this trial is 80%. If 80% (56/70) of participants are retained at end-of-study, the retention goal for this trial will be achieved. Previous studies by the study team had similar recruitment and retention rates of older participants.[^71-73^](#_ENREF_71) We are confident in our ability to recruit and retain the study participants necessary to conduct the proposed study. Participants will be determined to be adherent if they attend ≥80% of the intervention sessions (in-person meetings with study staff and online intervention sessions).[^71^](#_ENREF_71)^,^[^74^](#_ENREF_74)^,^[^75^](#_ENREF_75) Overall adherence and retention rates and associated 95% confidence intervals will be reported at baseline, end-of-study, and 3-month follow-up.

*Aim 2*: The primary outcome of the SPPB will be assessed at three time points: baseline, end-of-study, and 3-month follow-up. Efficacy will be based on 12 weeks. The SPPB score is a continuous variable, collected as the total score ranging from 0-12. Group differences in SPPB scores over the time will be tested using repeated measures ANOVA (SAS PROC MIXED). Using the planned contrasts command, we will investigate the changes in SPPB scores within each group, and between the intervention and control groups at end-of-study. Descriptive statistics and frequency distributions will be used to characterize the sample. To examine the effect of the intervention on the changes in physical function, a linear mixed effect model will be adopted using SAS PROC MIXED procedure. The intervention group (intervention vs. control) and time points (baseline, end-of-study, 3-month follow-up) serve as the categorical predictors, together with the intervention*time interaction term. A significant intervention*time interaction effect would indicate a differential change pattern over the time between the groups. Using post-hoc contrasts, we will compare group differences in all collected variables at each time point. Using planned contrasts command, we will investigate for each group the change from baseline to end-of-study, from end-of-study to 3-month follow-up, and from baseline to 3-month follow-up to capture the change patterns over the phases and Bonferroni correction will be applied. Preliminary effect sizes will be generated to inform future trials. A Shapiro-Wilk test, skewness, kurtosis, and Q-Q plots will be used to assess normality of survivor baseline and end-of-study outcomes. Based on normality tests, change in participant outcome measures from baseline to end-of-study will be assessed using paired t-tests or Wilcoxon’s signed rank tests.[^76^](#_ENREF_76) Analysis will be carried out using intent-to-treat (all participants) and modified intent-to-treat (adherent participants only). All quantitative analyses will be performed using SAS version 9.4.

**REFERENCES**References

1. Miller KD, Nogueira L, Devasia T, et al. Cancer treatment and survivorship statistics, 2022. *CA: A Cancer Journal for Clinicians.* 2022;n/a(n/a):in press.

2. Chavan P, Kedia S, Yu X. Physical and functional limitations in US older cancer survivors. *Journal of Palliative Care & Medicine.* 2017;7(4).

3. Guida JL, Holt CL, Dallal CM, He X, Gold R, Liu H. Social relationships and functional impairment in aging cancer survivors: A longitudinal social network study. *The Gerontologist.* 2019;60(4):607-616.

4. Greysen SR, Stijacic Cenzer I, Boscardin WJ, Covinsky KE. Functional impairment: An unmeasured marker of medicare costs for postacute care of older adults. *Journal of the American Geriatrics Society.* 2017;65(9):1996-2002.

5. Hewitt M, Rowland JH, Yancik R. Cancer survivors in the United States: Age, health, and disability. *The Journals of Gerontology: Series A.* 2003;58(1):M82-M91.

6. Siddique A, Simonsick EM, Gallicchio L. Functional decline among older cancer survivors in the Baltimore longitudinal study of aging. *Journal of the American Geriatrics Society.* 2021;69(11):3124-3133.

7. Williams GR, Chen Y, Kenzik KM, et al. Assessment of sarcopenia measures, survival, and disability in older adults before and after diagnosis with cancer. *JAMA Network Open.* 2020;3(5):e204783-e204783.

8. Kenzik KM, Morey MC, Cohen HJ, Sloane R, Demark-Wahnefried W. Symptoms, weight loss, and physical function in a lifestyle intervention study of older cancer survivors. *Journal of Geriatric Oncology.* 2015;6(6):424-432.

9. Cohen HJ. Keynote comment: cancer survivorship and ageing--a double whammy. *The Lancet Oncology.* 2006;7(11):882.

10. Leach CR, Bellizzi KM, Hurria A, Reeve BB. Is it my cancer or am I just getting older?: Impact of cancer on age-related health conditions of older cancer survivors. *Cancer.* 2016;122(12):1946-1953.

11. Petrick JL, Reeve BB, Kucharska-Newton AM, et al. Functional status declines among cancer survivors: trajectory and contributing factors. *Journal of Geriatric Oncology.* 2014;5(4):359-367.

12. Koll TT, Semin JN, Brodsky R, et al. Health-related and sociodemographic factors associated with physical frailty among older cancer survivors. *Journal of Geriatric Oncology.* 2021;12(1):96-101.

13. Studenski S, Perera S, Patel K, et al. Gait speed and survival in older adults. *JAMA.* 2011;305(1):50-58.

14. Brown JC, Harhay MO, Harhay MN. Physical function as a prognostic biomarker among cancer survivors. *British journal of cancer.* 2015;112(1):194-198.

15. Kline RM, Arora NK, Bradley CJ, et al. Long-term survivorship care after cancer treatment - Summary of a 2017 National Cancer Policy Forum Workshop. *Journal of the National Cancer Institute.* 2018;110(12):1300-1310.

16. Williams GR, Dunham L, Chang Y, et al. Geriatric assessment predicts hospitalization frequency and long-term care use in older adult cancer survivors. *Journal of Oncology Practice.* 2019;15(5):e399-e409.

17. Brown JC, Harhay MO, Harhay MN. Patient-reported versus objectively-measured physical function and mortality risk among cancer survivors. *Journal of Geriatric Oncology.* 2016;7(2):108-115.

18. Paddon-Jones D, Rasmussen BB. Dietary protein recommendations and the prevention of sarcopenia. *Curr Opin Clin Nutr Metab Care.* 2009;12(1):86-90.

19. Bloom I, Shand C, Cooper C, Robinson S, Baird J. Diet quality and sarcopenia in older adults: a systematic review. *Nutrients.* 2018;10(3):308.

20. Robinson S, Granic A, Sayer AA. Nutrition and muscle strength, as the key component of sarcopenia: An overview of current evidence. *Nutrients.* 2019;11(12):2942.

21. Silva Neto LS, Karnikowiski MG, Tavares AB, Lima RM. Association between sarcopenia, sarcopenic obesity, muscle strength and quality of life variables in elderly women. *Revista brasileira de fisioterapia (Sao Carlos (Sao Paulo, Brazil)).* 2012;16(5):360-367.

22. Kiwata JL, Dorff TB, Schroeder ET, et al. A pilot randomised controlled trial of a periodised resistance training and protein supplementation intervention in prostate cancer survivors on androgen deprivation therapy. *BMJ Open.* 2017;7(7):e016910.

23. Madzima TA, Ormsbee MJ, Schleicher EA, Moffatt RJ, Panton LB. Effects of resistance training and protein supplementation in breast cancer survivors. *Medicine & Science in Sports & Exercise.* 2017;49(7):1283-1292.

24. Stacey FG, James EL, Chapman K, Courneya KS, Lubans DR. A systematic review and meta-analysis of social cognitive theory-based physical activity and/or nutrition behavior change interventions for cancer survivors. *Journal of Cancer Survivorship.* 2015;9(2):305-338.

25. Demark-Wahnefried W, Morey MC, Sloane R, et al. Reach out to enhance wellness home-based diet-exercise intervention promotes reproducible and sustainable long-term improvements in health behaviors, body weight, and physical functioning in older, overweight/obese cancer survivors. *Journal of Clinical Oncology.* 2012;30(19):2354-2361.

26. Furness K, Sarkies MN, Huggins CE, Croagh D, Haines TP. Impact of the method of delivering electronic health behavior change interventions in survivors of cancer on engagement, health behaviors, and health outcomes: Systematic review and meta-analysis. *Journal of Medical Internet Research.* 2020;22(6):e16112.

27. Hong YA, Goldberg D, Ory MG, et al. Efficacy of a mobile-enabled web app (iCanFit) in promoting physical activity among older cancer survivors: a pilot study. *JMIR cancer.* 2015;1(1):e7.

28. Chan JM, Van Blarigan EL, Langlais CS, et al. Feasibility and Acceptability of a Remotely Delivered, Web-Based Behavioral Intervention for Men With Prostate Cancer: Four-Arm Randomized Controlled Pilot Trial. *J Med Internet Res.* 2020;22(12):e19238.

29. Bantum EOC, Albright CL, White KK, et al. Surviving and thriving with cancer using a Web-based health behavior change intervention: randomized controlled trial. *Journal of medical Internet research.* 2014;16(2):e54.

30. Allicock M, Kendzor D, Sedory A, et al. A pilot and feasibility mobile health intervention to support healthy behaviors in african american breast cancer survivors. *Journal of Racial and Ethnic Health Disparities.* 2021;8(1):157-165.

31. Roberts AL, Fisher A, Smith L, Heinrich M, Potts HWW. Digital health behaviour change interventions targeting physical activity and diet in cancer survivors: a systematic review and meta-analysis. *J Cancer Surviv.* 2017;11(6):704-719.

32. Blair CK, Harding E, Wiggins C, et al. A Home-Based Mobile Health Intervention to Replace Sedentary Time With Light Physical Activity in Older Cancer Survivors: Randomized Controlled Pilot Trial. *JMIR Cancer.* 2021;7(2):e18819.

33. Gorzelitz JS, Stoller S, Costanzo E, et al. Improvements in strength and agility measures of functional fitness following a telehealth-delivered home-based exercise intervention in endometrial cancer survivors. *Supportive Care in Cancer.* 2022;30(1):447-455.

34. Winters-Stone KM, Boisvert C, Li F, et al. Delivering exercise medicine to cancer survivors: has COVID-19 shifted the landscape for how and who can be reached with supervised group exercise? *Supportive Care in Cancer.* 2022;30(3):1903-1906.

35. Clinton SK, Giovannucci EL, Hursting SD. The world cancer research fund/American institute for cancer research third expert report on diet, nutrition, physical activity, and cancer: impact and future directions. *The Journal of nutrition.* 2020;150(4):663-671.

36. Siegel RL, Miller KD, Fuchs HE, Jemal A. Cancer statistics, 2022. *CA: a cancer journal for clinicians.* 2022.

37. Schmid D, Leitzmann M. Association between physical activity and mortality among breast cancer and colorectal cancer survivors: a systematic review and meta-analysis. *Annals of oncology.* 2014;25(7):1293-1311.

38. Demark-Wahnefried W, Cases MG, Cantor AB, et al. Pilot randomized controlled trial of a home vegetable gardening intervention among older cancer survivors shows feasibility, satisfaction, and promise in improving vegetable and fruit consumption, reassurance of worth, and the trajectory of central adiposity. *Journal of the Academy of Nutrition and Dietetics.* 2018;118(4):689-704.

39. Buffart LM, Kalter J, Sweegers MG, et al. Effects and moderators of exercise on quality of life and physical function in patients with cancer: An individual patient data meta-analysis of 34 RCTs. *Cancer Treatment Reviews.* 2017;52:91-104.

40. Cases MG, Frugé AD, Jennifer F, et al. Detailed methods of two home-based vegetable gardening intervention trials to improve diet, physical activity, and quality of life in two different populations of cancer survivors. *Contemporary clinical trials.* 2016;50:201-212.

41. Harris PA, Taylor R, Thielke R, Payne J, Gonzalez N, Conde JG. Research electronic data capture (REDCap)—a metadata-driven methodology and workflow process for providing translational research informatics support. *Journal of biomedical informatics.* 2009;42(2):377-381.

42. Harris PA, Taylor R, Minor BL, et al. The REDCap consortium: Building an international community of software platform partners. *Journal of biomedical informatics.* 2019;95:103208.

43. Rock CL, Thomson CA, Sullivan KR, et al. American Cancer Society nutrition and physical activity guideline for cancer survivors. *CA: A Cancer Journal for Clinicians.* 2022.

44. US Department of Agriculture and US Department of Health and Human Services. *Dietary Guidelines for Americans, 2020-2025.* Washington, DC: US Department of Agriculture and US Department of Health and Human Services;2020.

45. Weijs PJM, Wolfe RR. Exploration of the protein requirement during weight loss in obese older adults. *Clinical Nutrition.* 2016;35(2):394-398.

46. Prado CM, Cushen SJ, Orsso CE, Ryan AM. Sarcopenia and cachexia in the era of obesity: clinical and nutritional impact. *Proceedings of the Nutrition Society.* 2016;75(2):188-198.

47. Prado CM, Purcell SA, Laviano A. Nutrition interventions to treat low muscle mass in cancer. *Journal of Cachexia, Sarcopenia and Muscle.* 2020;11(2):366-380.

48. American College of Sports Medicine. American College of Sports Medicine position stand. Progression models in resistance training for healthy adults. *Medicine and science in sports and exercise.* 2009;41(3):687-708.

49. Focht BC, Lucas AR, Grainger E, et al. Effects of a group-mediated exercise and dietary intervention in the treatment of prostate cancer patients undergoing androgen deprivation therapy: Results From the IDEA-P trial. *Annals of Behavioral Medicine.* 2018;52(5):412-428.

50. Lucas AR, Focht BC, Cohn DE, Buckworth J, Klatt MD. A mindfulness-based lifestyle intervention for obese, inactive endometrial cancer survivors: a feasibility study. *Integrative Cancer Therapies.* 2017;16(3):263-275.

51. Focht BC, Rejeski WJ, Hackshaw K, et al. The Collaborative Lifestyle Intervention Program in Knee Osteoarthritis Patients (CLIP-OA) trial: Design and methods. *Contemporary Clinical Trials.* 2022;115:106730.

52. Schmitz KH, Courneya KS, Matthews C, et al. American college of sports medicine roundtable on exercise guidelines for cancer survivors. *Medicine & Science in Sports & Exercise.* 2010;42(7):1409-1426.

53. Chodzko-Zajko WJ, Proctor DN, Singh MAF, et al. Exercise and physical activity for older adults. *Medicine & science in sports & exercise.* 2009;41(7):1510-1530.

54. Ostir GV, Volpato S, Fried LP, Chaves P, Guralnik JM. Reliability and sensitivity to change assessed for a summary measure of lower body function: results from the Women's Health and Aging Study. *Journal of Clinical Epidemiology.* 2002;55(9):916-921.

55. Schulz KF, Altman DG, Moher D. CONSORT 2010 statement: updated guidelines for reporting parallel group randomised trials. *Trials.* 2010;11(1):1-8.

56. National Cancer Institute. *Diet History Questionnaire III.* Washington, DC: U.S. Department of Health and Human Services;2018.

57. Hays RD, Sherbourne CD, Mazel RM. The RAND 36‐item health survey 1.0. *Health economics.* 1993;2(3):217-227.

58. Focht BC, Rejeski WJ, Hackshaw K, et al. The Collaborative Lifestyle Intervention Program in Knee Osteoarthritis Patients (CLIP-OA) trial: Design and methods. *Contemporary clinical trials.* 2022;115:106730.

59. Umstattd MR, Motl R, Wilcox S, Saunders R, Watford M. Measuring physical activity self-regulation strategies in older adults. *J Phys Act Health.* 2009;6 Suppl 1:S105-112.

60. Norman CD, Skinner HA. eHEALS: The eHealth Literacy Scale. *J Med Internet Res.* 2006;8(4):e27.

61. Bowen DJ, Kreuter M, Spring B, et al. How we design feasibility studies. *American Journal of Preventive Medicine.* 2009;36(5):452-457.

62. Leon AC, Davis LL, Kraemer HC. The role and interpretation of pilot studies in clinical research. *Journal of Psychiatric Research.* 2011;45(5):626-629.

63. Czajkowski SM, Powell LH, Adler N, et al. From ideas to efficacy: The ORBIT model for developing behavioral treatments for chronic diseases. *Health Psychology.* 2015;34(10):971.

64. Perera S, Mody SH, Woodman RC, Studenski SA. Meaningful change and responsiveness in common physical performance measures in older adults. *Journal of the American Geriatrics Society.* 2006;54(5):743-749.

65. Kwon S, Perera S, Pahor M, et al. What is a meaningful change in physical performance? Findings from a clinical trial in older adults (the LIFE-P study). *JNHA-The Journal of Nutrition, Health and Aging.* 2009;13(6):538-544.

66. Salerno EA, Gothe NP, Fanning J, Peterson LL, Colditz GA, McAuley E. Effects of a DVD-delivered randomized controlled physical activity intervention on functional health in cancer survivors. *BMC Cancer.* 2021;21(1):870.

67. Owusu C, Margevicius S, Nock NL, et al. A randomized controlled trial of the effect of supervised exercise on functional outcomes in older African American and non‐Hispanic White breast cancer survivors: Are there racial differences in the effects of exercise on functional outcomes? *Cancer.* 2022;128(12):2320-2338.

68. Porter Starr KN, Pieper CF, Orenduff MC, et al. Improved function With enhanced protein intake per meal: A pilot study of weight reduction in frail, obese older adults. *The journals of gerontology Series A, Biological sciences and medical sciences.* 2016;71(10):1369-1375.

69. Cox M, Basen-Engquist K, Carmack CL, et al. Comparison of internet and telephone interventions for weight loss among cancer survivors: randomized controlled trial and feasibility study. *JMIR Cancer.* 2017;3(2):e7166.

70. Strath SJ, Swartz AM, Parker SJ, Miller NE, Grimm EK, Cashin SE. A pilot randomized controlled trial evaluating motivationally matched pedometer feedback to increase physical activity behavior in older adults. *Journal of Physical Activity and Health.* 2011;8(s2):S267-S274.

71. Krok-Schoen JL, Shim R, Nagel R, et al. Outcomes of a health coaching intervention delivered by medical students for older adults with uncontrolled type 2 diabetes. *Gerontology & Geriatrics Education.* 2017;38(3):257-270.

72. Krok-Schoen JL, Naughton MJ, Young GS, et al. Increasing adherence to adjuvant hormone therapy among patients with breast cancer: A smart phone app-based pilot study. *Cancer control : journal of the Moffitt Cancer Center.* 2019;26(1):1073274819883287.

73. Rosko AE, Huang Y, Jones D, et al. Feasibility of implementing an exercise intervention in older adults with hematologic malignancy. *Journal of Geriatric Oncology.* 2022;13(2):234-240.

74. van Dongen EJI, Haveman-Nies A, Doets EL, Dorhout BG, de Groot LCPGM. Effectiveness of a diet and resistance exercise intervention on muscle health in older adults: ProMuscle in practice. *Journal of the American Medical Directors Association.* 2020;21(8):1065-1072.e1063.

75. Algotar A, Hsu C-H, Chow HS, et al. Comprehensive lifestyle improvement program for prostate cancer (CLIPP): Protocol for a feasibility and exploratory efficacy study in men on androgen deprivation therapy. *JMIR Research Protocols.* 2019;8(2):e12579.

76. Rosner B, Glynn RJ, Lee MLT. The Wilcoxon signed rank test for paired comparisons of clustered data. *Biometrics.* 2006;62(1):185-192.
